# 2D4, a humanized monoclonal antibody targeting CD132, is a promising treatment for systemic lupus erythematosus

**DOI:** 10.1038/s41392-024-02017-6

**Published:** 2024-11-17

**Authors:** Huiqi Yin, Liming Li, Xiwei Feng, Zijun Wang, Meiling Zheng, Junpeng Zhao, Xinyu Fan, Wei Wu, Lingyu Gao, Yijing Zhan, Ming Zhao, Qianjin Lu

**Affiliations:** 1 Hospital for Skin Diseases, Institute of Dermatology, Chinese Academy of Medical Sciences and Peking Union Medical College, Nanjing, China; 2 Peking Union Medical College, Chinese Academy of Medical Sciences, Beijing, China; 3 Department of Dermatology, Second Xiangya Hospital, Central South University, Changsha, China

**Keywords:** Rheumatic diseases, Biologics, Immunological disorders

## Abstract

Current therapies for systemic lupus erythematosus that target a particular factor or cell type exhibit limited effectiveness. To address this limitation, our focus was on CD132, a subunit common to six inflammatory factor receptors implicated in SLE. Our study revealed heightened CD132 expression in SLE patients’ lymphocytes, contributing to the production of pro-inflammatory cytokines and immunoglobulins. We developed a novel humanized anti-CD132 monoclonal antibody, named as 2D4. 2D4 efficiently blocked IL-21 and IL-15, with limited effectiveness against IL-2, thereby suppressing T and B cells without disrupting immune tolerance. In the mouse immunization model, 2D4 virtually inhibited T cell-dependent, antigen-specific B-cell response. In lupus murine models, 2D4 mitigated inflammation by suppressing multiple pro-inflammatory cytokines and anti-dsDNA antibody titers, also diminishing proteinuria and glomerulonephritis. Compared to Belimumab, 2D4 exhibited superior efficacy in ameliorating the inflammatory state and preserving renal function. Moreover, 2D4 exhibited the ability to inhibit the production of pro-inflammatory factors and autoantibodies in PBMCs from individuals with SLE, highlighting its therapeutic potential for SLE individuals. Potent, 2D4 has the potential to significantly improve clinical outcomes in SLE and other complex autoimmune disorders.

## Introduction

Systemic lupus erythematosus (SLE) is a chronic autoimmune inflammatory condition that predominantly affects women in their childbearing age. Lupus nephritis (LN) emerges as a severe complication that develops in approximately half of all individuals with SLE, and it is associated with a noteworthy incidence of morbidity and mortality. Despite the administration of potent anti-inflammatory and immunosuppressive treatments, the development of chronic kidney disease due to LN remains a prevalent outcome. Within five years of onset, 5% to 25% of patients with proliferative LN encounter mortality directly attributed to renal disease.

B cells play a central role in thepathogenesis of SLE, primarily due to their capacity to present antigens to auto-reactive T cells, release inflammatory cytokines, and differentiate into antibody-secreting cells (ASCs), i.e., plasmablasts and plasma cells responsible for generating pathogenic autoantibodies.^1^ Consequently, targeting B cells and ASCs through depletion or inhibition emerges as a compelling therapeutic strategy for SLE and other autoimmune disorders. However, despite over two decades of research and more than 40 randomized clinical trials, only one such therapy, belimumab, has gained approval for SLE treatment, underscoring the intricate nature of the disease. Rituximab, which induces B cell depletion by binding to CD20, has been utilized for over 20 years in cases of refractory SLE and LN, despite not meeting primary endpoints in two phase III clinical trials. Belimumab, an anti-BAFF antibody, is approved for treating SLE and LN. Nevertheless, clinical remission, as assessed by Lupus Low Disease Activity State (LLDAS) or complete renal response, is achieved in only a minority of patients (12–14% or 30%, respectively).^2,3^ The demand for more effective agents persists. Recent evidence underscores the central role of T cells, including CD4 and CD8 subsets, in the pathogenesis of both SLE and LN. T cells mediate tissue damage and augment autoantibody production by fostering B cell differentiation, proliferation, and maturation. T cells constitute the majority of renal-infiltrating immune cells. Various CD4^+^ T helper (Th) cell subsets, including Th1, Th2, Th17 and T follicular helper (Tfh) cells, along with their associated cytokines, as well as cytotoxic CD8^+^ T cells, are implicated in the immune pathogenesis of both SLE and LN.^4^ For enhanced efficacy in SLE treatment, consideration of T cells is imperative.

CD132, also known as IL-2RG or common γ chain (γc) cytokine receptor, is the common subunit of γc cytokines, including interleukin-2 (IL-2), IL-4, IL-7, IL-9, IL-15, and IL-21. These cytokines trigger the activation of Janus kinases (JAKs), specifically JAK1 and JAK3. JAK1 associates with specific receptor subunits (namely IL-2RB, IL-4RA, IL-7R, IL-9R, and IL-21R), while JAK3 interacts with CD132, leading to JAK-induced phosphorylation,^5^ dimerization, and the translocation of STAT to the cell nucleus.^6,7^ γc cytokines exhibit broad pleiotropic effects on both T and B cells. IL-4 and IL-21 play pivotal roles in promoting B activation, proliferation, immunoglobulin class switching recombination, and plasma cell differentiation. The absence of IL-21 or IL-4 results in defective antibody and GC responses.^8,9^ Besides, IL-21 contributes to the differentiation of Th17 and Tfh cells. IL-15 and IL-21 actively participate in the generation and activity of cytotoxic CD8^+^ T cells (CTLs).^10,11^ IL-7 is essential for T cell development, while IL-9 has been identified as a late T cell growth factor.^12^

γc cytokines play intricate yet indispensable roles in the development of SLE. Elevated levels of IL-21 in SLE plasma, and polymorphisms in IL-21/IL-21R, have been correlated with SLE development.^13^ Evidence has demonstrated that IL-21 promotes autoimmune disorders in SLE by affecting the T and B cells.^14,15^ Increased serum IL-15 is implicated in SLE pathogenesis, particularly in LN.^16^ IL-15 promotes the formation and activation of CD8^+^ resident memory T cells (TRM) in the kidney, exacerbating podocyte injury and glomerulosclerosis.^17^ IL-4’s role in rescuing B cells from apoptosis is proposed to foster the survival of autoreactive B lymphocyte survival in lupus mouse models.^18^ In SLE patients, heightened IL-9 levels are observed, and successful disease control correlates with reduction in IL-9 concentrations. Soluble IL-7R levels correlate with SLE disease activity and anti-double-stranded DNA (anti-dsDNA) antibody concentration.^19^ IL-7 contributes to Th17 cell polarization in SLE, and the IL-7/IL-7R pathway destabilizes, creating conditions that override endogenous checkpoints against autoimmunity.^20^

In this study, we found the methylation of CD132 promoter region was significantly decreased in peripheral blood mononuclear cells (PBMC) from SLE patients, accompanied by a significant increase in CD132 expression in lymphocytes. Meanwhile, given the pivotal involvement of γc cytokines in lymphocytes function and the pathogenesis of SLE, a promising therapeutic avenue involves modulating their activities by targeting CD132. This approach holds potential for addressing the underlying mechanisms of the disease. However, it’s noteworthy that among γc cytokines, IL-2 differs in its role, as demonstrated in several studies where it plays a protective function in SLE. Deficient IL-2 production contributes to the immune system observed in SLE.^21^ Notably, low-dose IL-2 has shown efficacy and tolerability in SLE treatment by fostering regulatory T (Treg) cells and inhibiting Th17 and Tfh cells.^22–24^ To optimize the treatment of SLE, it is desirable to minimize the inhibition of IL-2 when targeting CD132. This strategy aims to maintain the beneficial impact on Treg cells, ensuring a balanced modulation of the immune response for improved therapeutic outcomes in SLE management. Our study is the first to investigate the therapeutic effects of targeting CD132 for the treatment of SLE, identifying a novel potential target for the therapeutic strategies of SLE. In addition, our data supports that 2D4 is a promising candidate molecule for future clinical trials of SLE.

## Results

### Elevated CD132 signaling in lymphocytes of SLE patients contributes to pro-inflammatory responses

**Fig. 1:**
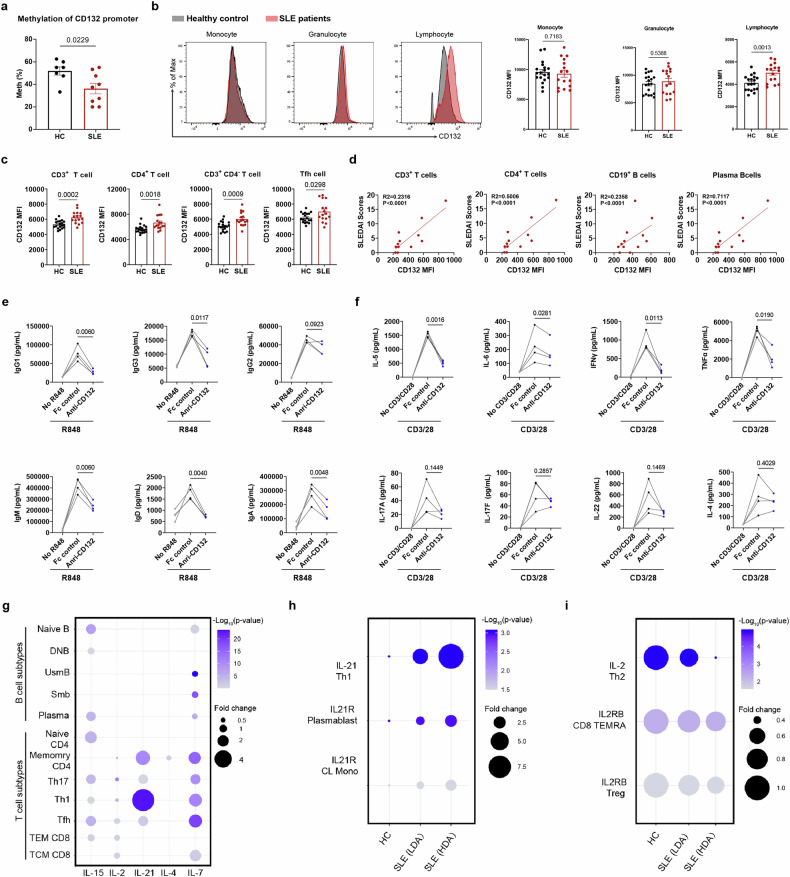
Elevated CD132 signaling in lymphocytes of SLE patients contributes to pro-inflammatory responses. **a** Methylation of CD132 promoter in PBMCs from healthy control (HC) and SLE patients (SLE) (*n* ≥ 7). **b** Analysis of mean fluorescence intensity (MFI) of CD132 in PBMCs from HC and SLE by flow cytometry (*n* ≥ 16). **c** Flow cytometric analysis of CD132 MFI in T cell subtypes from HC and SLE (*n* ≥ 16). **d** Correlation analysis of CD132 MFI with SLEDAI scores in CD3^+^ T, CD4^+^ T, CD19^+^ B and plasma cells of SLE PBMCs (*n*= 12). Two-tailed Spearman correlation test was used. **e** CD19^+^ B cells from healthy donors were in vitro stimulated with R848, in the presence or not of Anti-CD132 (REGN7257) or a control antibody (Fc control), and immunoglobulins release was detected by CBA at day 4. **f** CD4^+^ T cells from healthy donors were in vitro stimulated with anti-CD3 and anti-CD28 antibodies, in the presence or not of Anti-CD132 or Fc control, and cytokines release was detected by CBA at day 3. **g** Identification of differentially expressed genes of γc cytokines between HC and SLE in different lymphocyte subtypes (*p* < 0.05) by reanalyzing a published data, which was deposited in the National Bioscience Database Center Human Database with the accession number of E-GEAD-397. (h-i) mRNA expression of IL-21 and IL-2 in different lymphocyte subtypes of HC, SLE with low disease activity (LDA), and SLE with high disease activity (HDA) in a published data, which was deposited in the National Bioscience Database Center Human Database with the accession number of JGAS000220. Symbols represent individual persons. Error bars represent ±SEM. Student’s *t*-tests (**a**, **b**, **c**, **e** and **f**) were used. In **b**, **c**, data were pooled from 2 experiments. In **e**–**f**, data represent of 4 (**e**) or 3 (**f**) independent experiments

We identified a significant decrease in methylation levels of CD132 promoter in PBMCs from SLE patients (Fig. 1a). Then we detected the CD132 expression in different immune subtypes in the peripheral blood by flow cytometry. No significant changes in CD132 expression in monocytes and granulocytes of SLE were observed (Fig. 1b). In contrast, lymphocytes from SLE patients exhibited a notable upregulation of CD132 expression across almost all lymphocyte subtypes, including CD3^+^ T cell and CD19^+^ B cell subtypes (Fig. 1c and supplementary Fig. 1b). However, initially we did not find that CD132 expression differed among patients with different disease activity, perhaps due to most of these patients were being treated with medication. To avoid interference from therapeutic drugs, we screened and resuscitated twelve PBMC samples of untreated patients with different SLEDAI from our sample bank and found that the expression of CD132 was significantly and positively correlated with SLEDAI scores in CD3^+^ T, CD4^+^ T, CD19^+^ B and plasma cells (Fig. 1d).

To explore the immunomodulatory role of upregulated CD132 in SLE disease, we purified CD4^+^ T cells and CD19^+^ B cells for probing the effects of CD132 blockade, using REGN7257, a pan CD132 blocking mAB.^5^ We interrogated the role of CD132 signaling in antibodies secreting by human B cells firstly. In vitro cultures of isolated human B cells revealed that R848 stimulation led to robust antibodies production, with CD132 signaling inhibition substantially reducing the levels of IgG1, IgG2, IgG3, IgM, IgA, and IgD, without affecting cell viability (Fig. 1e and supplementary Fig. 1d). Purified human CD4^+^ T cells stimulated with anti-CD3 and anti-CD28 antibodies exhibited robust production of lupus-related pro-inflammatory cytokines (e.g., IFN-γ, TNF-α, IL-5, and IL-6) upon anti-CD3/CD28 beads stimulation. However, these cytokines were effectively suppressed following CD132 blockade with REGN7257, without affecting cell viability (Fig. 1f and supplementary Fig. 1f).

The immunomodulatory role of CD132 is inseparable from its six ligands, γ chain cytokines. To elucidate the role of γ chain cytokines in SLE, we analyzed a published single-cell transcriptome data, which covers 27 immune cell types from 62 SLE and 79 healthy donors.^25^ IL-21, IL-15, and IL-7 exhibited significant increases in several T and B cell subtypes of SLE, including Naive B, Naive CD4^+^ T, Memory CD4^+^ T, Th17, Tfh, Th1 and CD8^+^ TCM cells, among which IL-21 increased most significantly (Fig. 1g). In contrast, IL-2 demonstrated a substantial decrease in several immune cells of SLE (Fig. 1g), consistent with the different roles of γ chain cytokines in SLE pathologies.

### 2D4 is a potent IL-21 antagonist with limited impact on IL-2 compared to REGN7257 in targeting γc cytokines

To better manipulate CD132 for treating SLE, we conducted a reanalysis on another large-scale transcriptome dataset that included SLE patients with different disease activities.^26^ The results revealed a significantly elevated expression of IL-21 and its receptor in patients with low disease activity (LDA), with even higher levels in those with high disease activity (HDA) (Fig. 1h). Conversely, IL-2 and its receptor showed a different pattern, with significantly lower expression in patients with LDA and further reduction in those with HDA (Fig. 1i). These suggest that IL-21 and IL-2 are significantly associated with SLE disease status and activity. Considering the protective properties of IL-2 against SLE disease and the high expression and pathogenic role of IL-21,^27,28^ we used IL-2 and IL-21 as the primary screening targets. We aimed to identify an anti-huCD132 with a superior IL-21 blocking activity but weaker IL-2 blocking activity than REGN7257. An initial screening on a monoclonal antibody library obtained from the phage display acquisition technique, using PBMCs from healthy volunteers, led to the identification of two molecules, 2D4 and 5H10, comparable to REGN7257 in blocking IL-21 (Supplementary Fig. 2a). We then compared the activity in blocking IL-2 and found that 5H10 was equal to REGN7257 in blocking IL-2 activity, whereas 2D4 was significantly weaker (Supplementary Fig. 2b).

**Fig. 2:**
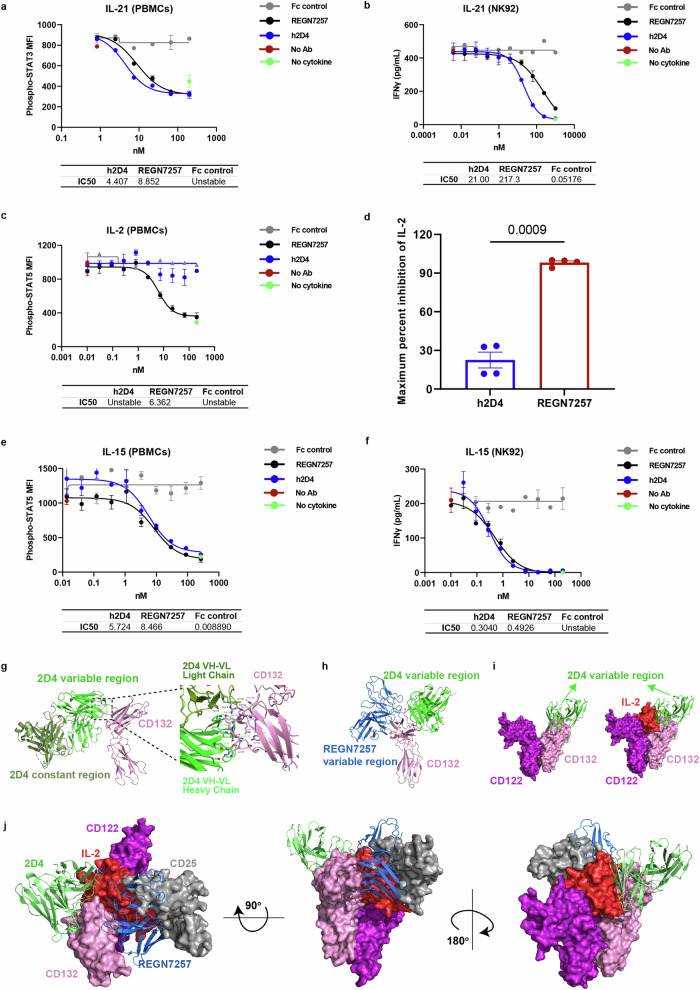
2D4 is an IL-21-enhanced and IL-2-weak antagonist of γc cytokines compared to REGN7257. **a**, **c**, **e** Human PBMCs were blocked with gradient dilutions of 2D4 or REGN7257 followed by stimulation with human IL-21, IL-2, or IL-15, inhibition of STATs phosphorylation (phospho-STAT3 or phospho-STAT5) in CD3^+^ T cells was assessed by flow cytometry. **d** Human PBMCs were blocked with gradient dilutions of 2D4 or REGN7257 followed by stimulation with human IL-2 and maximum percent inhibition of STAT5 phosphorylation in CD3^+^T cells by 2D4 and REGN7257 in another 4 healthy donors. The maximum percent inhibition of IL-2 was determined using the following formula: (MFI (No Ab)-MFI value corresponding to 200 nm antibody)/(MFI (No Ab)-MFI (No cytokine)) × 100%. **b**, **f** NK92 cell lines were blocked with gradient dilutions of 2D4 or REGN7257 followed by stimulation with human IL-21 or IL-15, inhibition of IFN-γ release was assessed by ELISA. **g** Ribbon representation of the cryoEM structure of 2D4 in complex with CD132 and interface analysis between the variable domain (green) of 2D4 Fab and CD132 (pink). Residues on the interface were represented as sticks (main chain or side chain), where oxygen and nitrogen atoms were colored red and blue, respectively. Residues with labels shown in the figure were selected based on the interaction pairs (interatomic distances <4 Å for hydrogen bonds/salt bridges, <5 Å for hydrophobic interactions). **h** Superposition of the CD132-2D4 complex structure with PDB:8EPA. The constant region of 2D4 was omitted for clarity. **i** Superposition of the CD132-2D4 variable structure and PDB: 2ERJ, CD25 and the constant region of 2D4 were not shown for clarity. **j** Superposition of the CD132-2D4 complex structure, PDB: 8EPA and PDB:2ERJ. CD132 (pink), 2D4 (green), REGN7157 (blue), lL-2 (red), CD122 (purple), CD25 (grey). The constant region of 2D4 was omitted for clarity. In d, data were pooled from 3 experiments and student’s *t*-test was used. Data are representative of 5 (**a**, **b**) or 3 (**e**, **f**) independent experiments

We fully humanized 2D4 and further fine-tuned the blocking activities of h2D4 and REGN7257 against IL-2, IL-7, IL-15, and IL-21 in a natural human lymphocyte cell line (NK92) and PBMCs of healthy donors. In the PBMCs validation system, we found that 2D4 had a > 2-fold lower median inhibition concentration (IC_50_) value relative to REGN7257 in blocking IL-21 by examining the level of STAT3 phosphorylation in CD3^+^ T cells downstream of CD132 (Fig. 2a). Surprisingly, in natural NK92 cells, 2D4 had a 10-fold better IC_50_value in blocking IL-21 activity relative to REGN7257 by assaying the level of IFN-γ secretion further downstream (Fig. 2b). These results confirmed that the blocking activity of 2D4 on IL-21 was superior to that of REGN7257. Regarding the blocking effect on IL-2, REGN7257 was still efficient enough to block the phosphorylation of STAT5 downstream of CD132 in PBMCs when the time of IL-2 stimulation increased to 24 h. Whereas 2D4 exhibited minimal blocking effects on IL-2 under the same conditions (Fig. 2c). The blocking ability of both molecules for IL-2 was repeated in another four healthy donors (Supplementary Fig. 2c), confirming that REGN7257 had a 100% maximum inhibition of IL-2, whereas 2D4 had only about 25% (Fig. 2d). In the system of IFN-γ secretion by NK92, while a high concentration of REGN7257 could block IFN-γ secretion completely, 2D4 could not achieve complete blockage (Supplementary Fig. 2d), validating that 2D4 is weaker than REGN7257 in blocking IL-2 activity. The NK92 cell line used is an IL-2-dependent type. Its daily culture requires the addition of IL-2, making the cells much more sensitive than PBMCs to IL-2. The concentration of IL-2 used to stimulate PBMCs is 100 times higher than that required to activate NK92. Thus, 2D4 demonstrated a more significant inhibitory effect inside NK92 than PBMCs. For IL-15 blocking, 2D4 displayed a slightly better IC_50_ than REGN7257 (Fig. 2e, f). The effect of 2D4 and REGN7257 on IL-4 in PBMCs, evaluated through downstream CD23 expression, showed that neither had a strong inhibitory effect on IL-4 (Supplementary Fig. 2e). This is due to the relative complexity of the receptor complex for IL-4, which contains the type I receptor complex with CD132 and IL-4Rα, and the type II receptor complex containing IL-4Rα and IL-13Rα1. While blockade of IL-4Rα with CM310, which blocks IL-4Rα,^29^ completely blocks IL-4 stimulation-induced CD23 upregulation, whereas blockade of CD132 only partially inhibits it (Supplementary Fig. 2e). Furthermore, we tested the effect of 2D4 on IL-7 and IL-9 in PBMCs and M07E cell lines, respectively, revealing that 2D4 is less effective in blocking IL-7 and IL-9 (Supplementary Fig. 2f–g).

To investigate the molecular mechanism underlying the differential effects of blocking IL-2 activity by 2D4 and REGN7257, we utilized cryo-electron microscopy (cryo-EM) to resolve the structure of 2D4 antibody in complex with the CD132 antigen. The structure revealed that 2D4 binds to the D1 domain of the type III structural region of the CD132 extracellular fibronectin, but its binding site is entirely distinct from that of REGN7257, located on the opposite side of CD132 D1 (Fig. 2g, h). To illustrate the spatial hindrance between the Fabs and the IL-2 complex, we superimposed the CD132 chain from the structures of 2D4-CD132, PDB: 8EPA, and PDB: 2ERJ. Compared to REGN7257, the spatial hindrance between 2D4 and IL-2 is significantly reduced, with no overlap between the positions of 2D4 and CD25 (lL2Ra). In contrast, in the case of PDB: 8EPA, REGN7257 not only conflicts with IL-2, but also shows substantial overlaps with CD25 (Fig. 2i, j). This observation correlates with the functional assay results, where the inhibitory effect of 2D4 on IL-2 was not as potent as that of REGN7257.

### 2D4 suppresses T and B cells without affecting the proportion of Treg

2D4 is fully humanized and ineffectual in mouse CD132 binding. To understand the in vivo impact of 2D4 on lymphocyte populations, we genetically modified CD132^hu/hu^ mice by replacing the mouse *Cd132* DNA sequence with its corresponding human sequence (Supplementary Fig. 3a). Peripheral blood analysis revealed no significant differences in leukocyte, erythrocyte, lymphocyte, monocyte, and neutrophil counts but a slight increase in platelets in CD132^hu/hu^ mice compared to wild-type (WT) mice (Supplementary Fig. 3b). Flow cytometric analysis demonstrated only a slight decrease in CD4^+^ T and NK cell count in CD132^hu/hu^ mice, with other cell types, including various CD8^+^ T cells, CD19^+^ B cells and NK cells, maintaining normal counts overall (Supplementary Fig. 3c). CD132 signaling pathways in splenocytes from both CD132^hu/hu^ and wild-type mice were able to be activated by all mouse γc cytokines (Supplementary Fig. 3d). 2D4 and REGN7257 are IgG4 subclasses monoclonal antibodies that lack Fc effector function, and therefore do not mediate ADCC or CDC effects (Supplementary Fig. 3e, f).

**Fig. 3:**
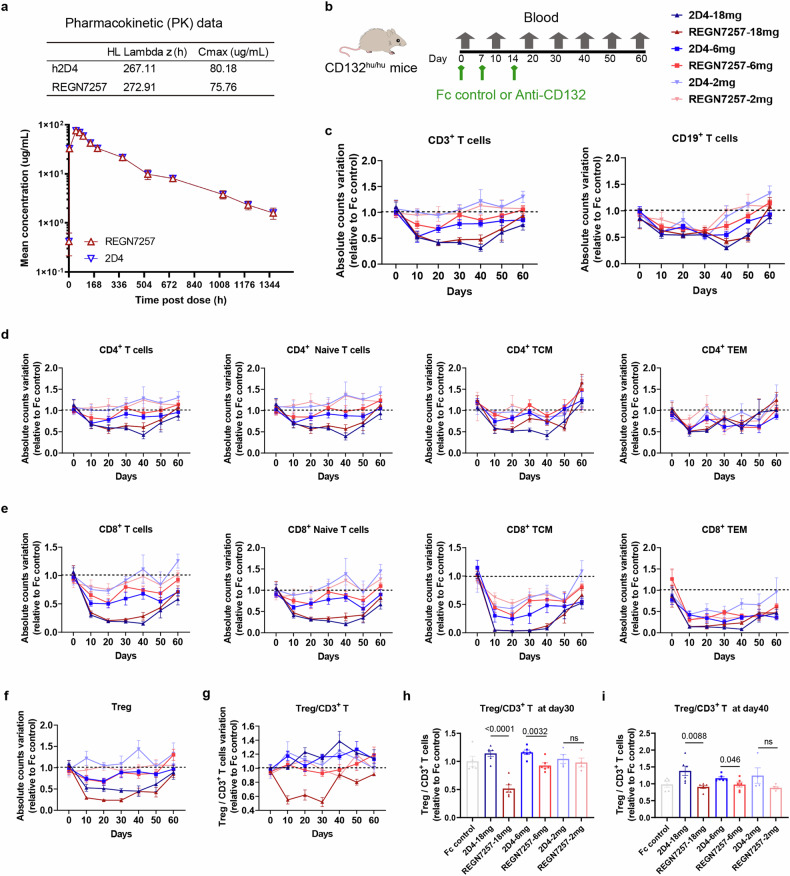
2D4 suppresses T and B cells without disturbing Treg proportion in T cells in vivo. **a** B-hFcRn mice (*n* = 4) were administered a single dose of 2D4 or REGN7257 (10 mg/kg) intravenous infusion on day 0. Serum was collected at various time points (before infusion and at 0.083, 4, 48, 72, 96, 144, 192, 360, 582, 696, 1032, 1200, and 1368 hours post infusion), and samples were analyzed for test article concentrations using ELISA. Dose-normalized concentration curves are shown. Cmax = maximum concentration; HL Lambda Z= Terminal Half-life. **b** CD132 ^hu/hu^ mice were dosed (days 0, 7, 14) with 2D4, REGN7257 or a Fc control, followed by a recovery period (*n* ≥ 4). **c** –**f** Absolute counts of T cell, B cell, and T cell subtypes in blood were analyzed over time by hematology analysis and flow cytometry, changes in the cell counts of the experimental group relative to the Fc control group over time were shown. **g**–**i** Treg/ CD3^+^ T cell ratio was analyzed over time by flow cytometry, and the results at day 30 and day 40 are shown separately in **h**, **i**. Symbols represent individual mice. Data are representative of 2 independent experiments

A pharmacokinetics study in humanized FcRn transgenic mouse (B-hFcRn mice) compared 2D4 and REGN7257 at a single 10 mg/kg intravenous (IV) dose, revealing comparable dose-normalized serum concentrations (Fig. 3a). In a pharmacodynamic study, CD132^hu/hu^ mice were injected with 2D4, REGN7257 or Fc control (Fig. 3b). Hematology analysis indicated a significant reduction in leukocyte counts, including T-lymphocytes and subtypes, B-lymphocytes, and NK-cells (Fig. 3c–e and Supplementary Fig. 3g, h). Dose-dependent effects were observed, with cell subsets recovering over time.

As IL-2 is critical for Treg cell homeostasis, we evaluated the impact of 2D4 and REGN7257 on Treg subpopulations. Both high dose 2D4 and REGN7257 reduced absolute Treg cell counts, but the inhibitory effect of REGN7257 was significantly stronger than that of 2D4 (Fig. 3f). More importantly, high-dose REGN7257 downregulated Treg proportions in CD3^+^ T cells, reaching its lowest level at day 30, while 2D4 had no effect on Treg proportions and even showed an up regulatory trend in later stages of recovery (Fig. 3g–i). In summary, 2D4 and REGN7257 exhibited comparable in vivo effectiveness. Importantly, 2D4 effectively inhibited lymphocytes without altering the proportion of Treg in T cells, preserving immune tolerance crucial for SLE patients.

### 2D4 suppresses T cell-dependent, antigen-specific B-cell response in germinal center

**Fig. 4:**
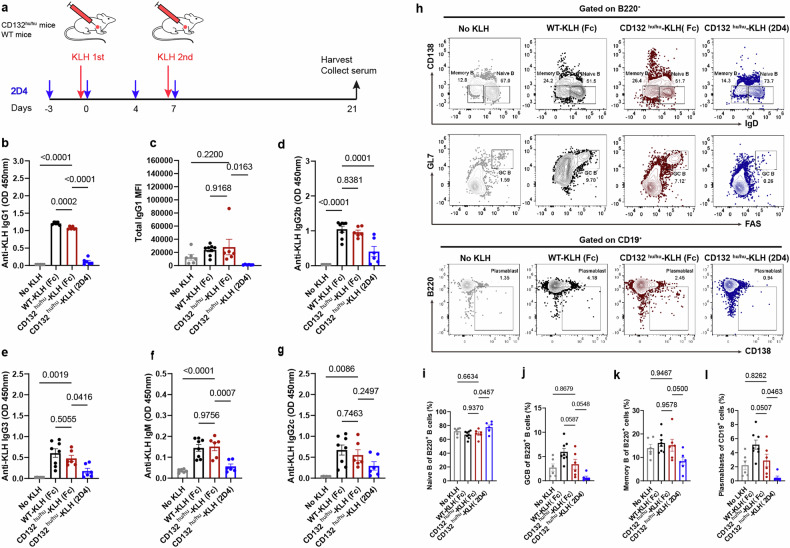
2D4 inhibits T cell–dependent antibody production in KLH–immunized mice. **a** CD132^hu/hu^ mice and WT mice were challenged with KLH on days 0 and 7, and CD132^hu/hu^ mice were dosed subcutaneously with 2D4 or Fc control (20 mg/kg) on days -3, 0, 4, and 7, and serum was collected, and mice were harvested on day21 (*n* ≥ 6). **b**, **d** –**g** Day 21 serum samples were analyzed for KLH-specific immunoglobulins by ELISA. **c** The total IgG1 level in serum at day 21 was analyzed by CBA. **h** Representative flow cytometry diagrams of B cell subtypes. The proportion of cells of B-cell subsets in dLNs at day 21 was determined by flow cytometry, including Naive B cells (**i**), GC B cells (**j**), memory B cells (**k**), and plasmablasts (**l**). Symbols represent individual mice. OD=optical density. Data are representative of 3 independent experiments

To further access the in vivo immuno-modulatory activity of 2D4 in vivo. CD132^hu/hu^ and WT mice were challenged with keyhole limpet hemocyanin (KLH) as a model antigen and treated with 2D4 to evaluate its effect on T cell-dependent, antigen-specific B-cell response (Fig. 4a). Flow cytometry and serum collection on day 21 post-immunization were employed for immune cell analysis. Serum antibody detection revealed a significant increase in KLH-specific IgG1 after immunization, with 2D4 fully inhibiting KLH-specific IgG1 production (Fig. 4b). IL-21 and IL-4 are crucial for the production of IgG1 isotype antibody and mice deficient in IL-4 or IL-21 signaling have lower IgG1 after immunization.^30,31^ We detected total IgG1 levels and found 2D4 markedly reduced total IgG1 levels (Fig. 4c). Besides, 2D4 significantly decreased the production of KLH-specific IgG2b, IgG3, and IgM, although there was only a downward trend for IgG2c (Fig. 4d–g).

Consistent with the reduction of antigen-specific immunoglobulins, germinal center (GC) response was efficiently reduced by 2D4, evident in the increased numbers of naive B cells and decreased accumulation of GC B cells (B220^+^ FAS^+^ GL-7^+^) in drainage lymph nodes (dLNs) of the 2D4 group (Fig. 4h–j). Moreover, the numbers of memory B (B220^+^ IgD^-^ CD138^-^) cells and plasmablasts (CD19^+^ B220^low^ CD138^+^) were reduced (Fig. 4k, l).

### 2D4 prevents inflammatory milieu in lupus mice by dual inhibition of T and B cells

**Fig. 5:**
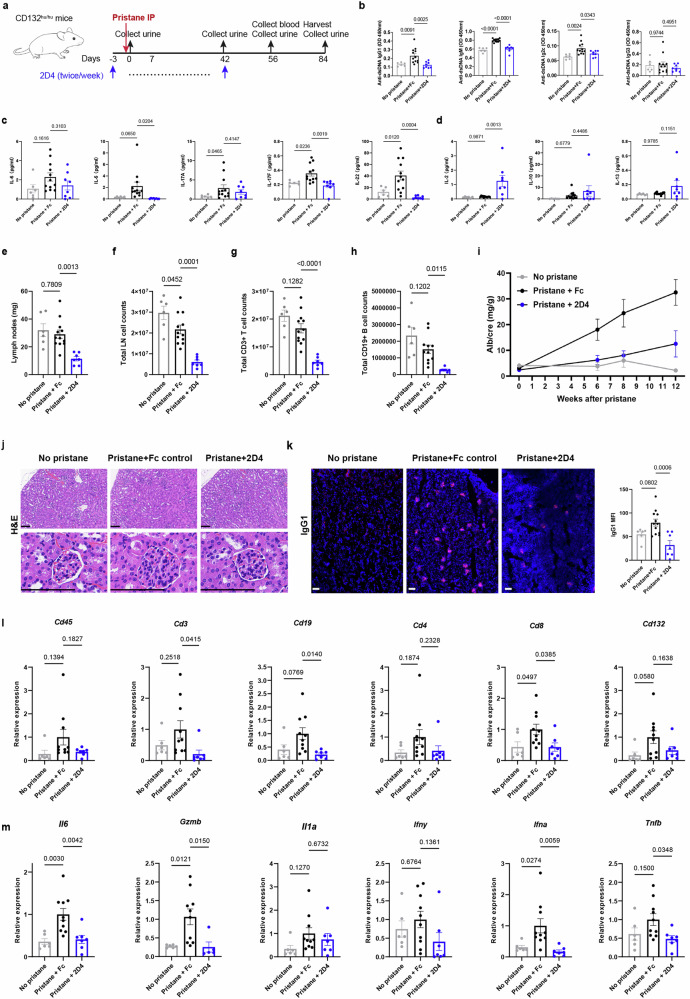
2D4 alleviates disease conditions in mouse models of SLE. **a** CD132^hu/hu^ mice were challenged with pristane on day 0 and received 20 mg/kg 2D4 or Fc control subcutaneously every 3–4 days from day -3 to day 42 for 15 total doses (*n* ≥ 6 animals per group). **b** Anti-dsDNA immunoglobulin levels in serum at 8 weeks after pristane were analyzed by ELISA. **c**, **d** Serum cytokine levels at 8 weeks after pristane.**e** Weight of dLNs. Populations of total cells (**f**), CD3^+^ T cells (**g**) and CD19^+^ B cells in dLNs (**h**). **i** Urinary Alb/cre was detected over time by ELISA. **j** H&E staining of kidney, scale bar = 100 μm. **k** Renal IgG1 deposit was evaluated by immunofluorescence staining (IF), and MFI was counted by Image J, scale bar = 200 μm. **l**, **m** Renal tissue mRNA expression of mouse immune genes and pro-inflammatory genes were measured by qPCR and expressed relative to mouse *Gapdh* mRNA expression. Data are representative of 2 independent experiments

2D4 was next evaluated in a pristane-induced mouse model of lupus, characterized by elevated inflammatory cytokines and autoantibodies in serum, eventually progressing to glomerulonephritis, which can mimic human SLE. In this study, 10-week-old CD132^hu/hu^ mice were randomized (according to the body weight) and treated prophylactically twice weekly with 2D4 or Fc control, followed by pristane challenge (Fig. 5a). Mouse serum collected at 8 weeks after pristane-challenge revealed increased levels of different isotypes of anti-dsDNA antibodies, with 2D4 significantly inhibiting IgG1, IgM and IgG2c (Fig. 5b). Proinflammatory cytokines, i.e., IL-5 and IL-17F, which play vital roles in SLE, were reduced by 2D4 treatment (Fig. 5c), while anti-inflammatory cytokines, i.e., IL-10 and IL-13 were not affected (Fig. 5d). These findings demonstrated that CD132 blockade with 2D4 improved the in vivo inflammatory environment of lupus mice. Besides, IL-2 level was evaluated robustly after 2D4 treatment, suggesting a potential protective role in SLE (Fig. 5d).

To investigate the mechanism of inhibition of secretion of inflammatory factors and autoantibodies, CD132-dependent cellular changes in the dLNs of pristane-induced lupus mice were analyzed using flow cytometry. The weight and total cell numbers of dLNs were decreased to about one-third of the control group (Fig. 5e, f), accompanied by significant reductions in the CD3^+^ T cells, CD19^+^ B cells, and all kinds of T cell subtypes (Fig. 5g, h and Supplementary Fig. 4a, b). In terms of B cells, not only the population of all B cell subtypes declined, but also the proportions of the B-cell subtypes significantly altered. The ratio of naive B was up-regulated considerably, and the proportions of memory B cells, GC B cells, and plasmablasts were significantly decreased (Supplementary Fig. 4c–i).

### 2D4 improves renal function in a pristane-induced mouse model of SLE

The urinary albumin to creatinine ratio (Alb/cre), a reliable indicator of kidney function, was significantly upregulated in the Fc control group at 6 weeks after pristane induction, indicating renal damage. In contrast, the ratio was significantly lower in 2D4-treated mice, only slightly elevated over disease-naive mice at 8 weeks after pristane administration (Fig. 5i). Histological analyses revealed glomerular changes such as glomerulomegaly, dilated glomerular capillaries, and narrowing of the glomerular capsule lumen in mice administered Fc control mice, whereas these changes were less pronounced in the 2D4-treated group (Fig. 5j). Furthermore, 2D4 reduced renal IgG1 deposits (Fig. 5k).

To assess renal inflammatory cell infiltration in pristane-induced SLE mice, we measured the expression of *Cd45*, *Cd3*, *Cd19*, *Cd4, and Cd8*, which indicate immune cell, T cell, B cell, Th cell, and CTL infiltration, respectively, using quantitative real-time PCR (qPCR). While these markers showed an upward after pristane induction in the Fc control group, only *Cd8* exhibited a statistically significant difference. Treatment with 2D4 significantly reduced the expression of CD3^+^ T cell, CD8^+^ T cell, and CD19^+^ B cells (Fig. 5l). *Cd132* expression increased in the Fc control group after pristane induction, indicating the role of CD132^+^ cells in renal tissue inflammation (Fig. 5l). Besides, the expression of lupus-related pro-inflammatory factors, including *Il6, Gzmb, and Ifna*, decreased with 2D4 treatment, the expression of *Tnfb* was also reduced (Fig. 5m). Collectively, these results indicate that 2D4 alleviated renal inflammation and protected mice from LN.

**Fig. 6:**
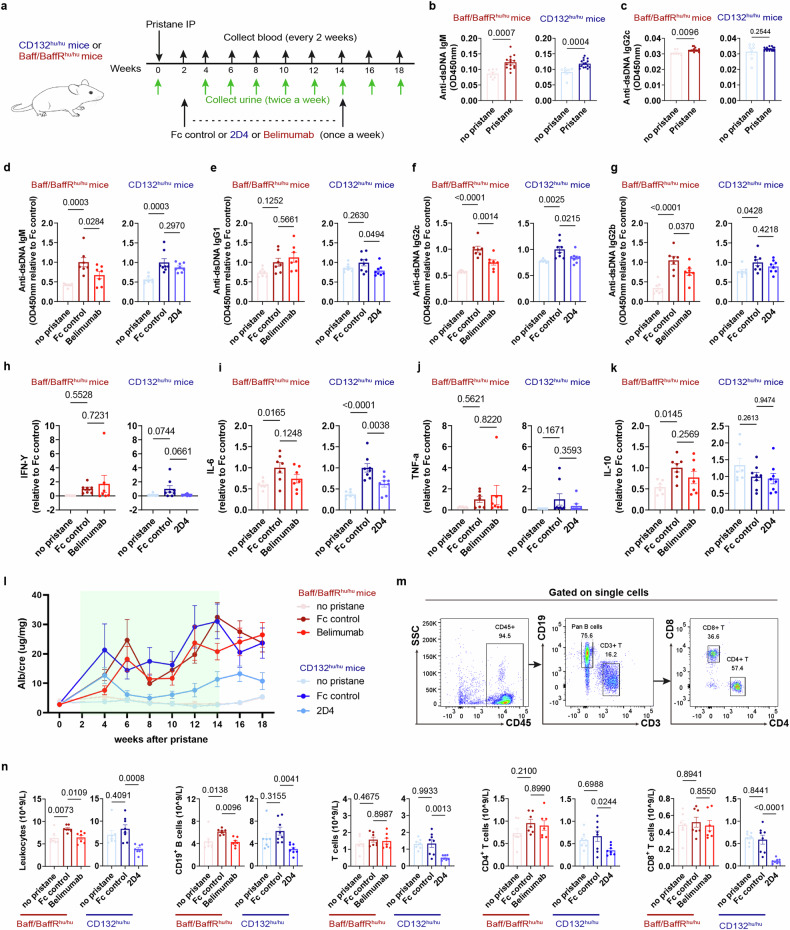
2D4 demonstrates enhanced efficacy than Belimumab in lupus nephritis. **a** BAFF/BAFFR^hu/hu^ mice and CD132^hu/hu^ mice were challenged with pristane on day 0 and received 10 mg/kg Belimumab or 2D4 respectively once a week from week 2 to week 14 for 13 total doses (*n* ≥ 7 animals per group). **b**, **c** Anti-dsDNA immunoglobulin levels in serum at 2 weeks after pristane were analyzed by ELISA. **d**–**g** Anti-dsDNA immunoglobulin levels at 14 weeks after pristane were analyzed by ELISA, OD450nm relative to Fc control was shown. **h**–**k** Serum cytokine levels at 14 weeks after pristane were analyzed by ELISA, cytokine level relative to Fc control was shown. **l** Urinary Alb/cre was detected over time by ELISA. **m**, **n** Gating strategy and population of lymphocyte subtypes in blood analyzed by hematology analysis and flow cytometry. In **b**–**k**, ELISA date of each figure was collected on the same plate at the same time to compare OD450-OD570 values

Belimumab, an anti-BAFF antibody, is a representative of B-cell-targeted therapy approved for treating SLE.^32^ Belimumab, targets only B cells, therefore, we hypothesized that 2D4 would be more effective in treating SLE than Belimumab. To test this hypothesis, we compared the efficacy of the two in a pristane-induced SLE mouse mode. 8-week-old CD132^hu/hu^ or BAFF^hu/hu^ /BAFFR^hu/hu^ (BAFF/BAFFR humanized) mice were randomized assigned and challenged with pristane. 2 weeks after pristane induction, the serum anti-dsDNA IgM and IgG2c were elevated, then the mice were treated once weekly with 2D4, Belimumab, or Fc control (Fig. 6a–c). Mouse serum was collected every 2 weeks after pristane. Autoantibodies at week 14 and cytokine levels at week 12 in serum were detected and shown. Among all the anti-dsDNA immunoglobulin subtypes, IgG1 and IgG2c were significantly inhibited by 2D4, whereas IgM, IgG2c and IgG2b were significantly inhibited by Belimumab treatment (Fig. 6d–g). Additionally, 2D4 also ameliorated the in vivo inflammatory state of the mice by inhibiting pro-inflammatory factors without affecting anti-inflammatory factors, consistent with Fig. 5c, whereas Belimumab did not affect these factors (Fig. 6h–k). We also examined the Alb/cre of the mice over time. Only 2D4 was able to significantly inhibit the elevation of the urinary Alb/cre (Fig. 6l). Moreover, we then examined their effects on circulating immune cells in mice by flow cytometry, and as expected, both Belimumab and 2D4 reduced circulating CD19^+^ B cells by a similar percentage. However, 2D4 was also effective in reducing circulating T cells, including CD4^+^ and CD8^+^ T cells, which were not affected by Belimumab (Fig. 6m, n). Therefore, we concluded that 2D4 was significantly more effective than Belimumab in treating LN by inhibiting both T and B cells.

### 2D4 suppresses the activities and functions of SLE T and B cells both ex vivo and in vivo

**Fig. 7:**
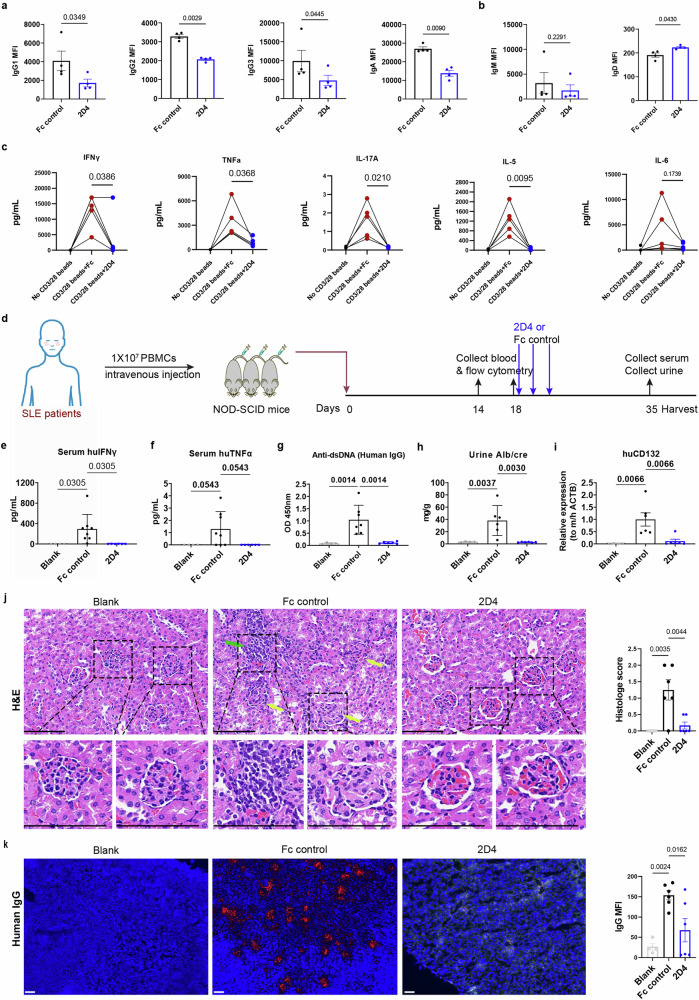
2D4 suppressed antibodies and cytokines secreted by PBMCs from SLE patients in both ex vivo and in vivo assays. **a**, **b** PBMCs from SLE patients were cultured in the presence of 20 μg/mL IgG4 isotype control or 2D4, cell supernatant was harvested, and human immunoglobulins release was detected by CBA at day 4 (*n* = 4). **c** PBMCs from SLE patients were cultured and stimulated with anti-CD3/CD28 beads, in the presence of 20 μg/mL IgG4 isotype control or 2D4, cell supernatant was harvested, and human cytokines release was detected by CBA at day 4. Each line represents a biological repetition (*n* = 5). **d** NOD-SCID mice were engrafted with 1×10^7^ SLE PBMCs or not (Blank) on day 0 and received 2D4 or Fc control subcutaneously on day 18 every 3–4 days for 3 total doses. Mice were euthanized at day 35 after SLE PBMC engraftment for serum and renal analysis (*n*≥ 4). **e**–**g** Serum cytokines and anti-dsDNA titers in serum were analyzed by ELISA. **h** Terminal Urinary Alb/cre. **i** Renal tissue mRNA expression of human CD132 was measured by qPCR, expression versus mouse/human β-actin (ACTB). **j** H&E staining of kidney and histology score as a sum of total glomerular, tubular, and interstitial lesions. The green arrow represents cell infiltration. Yellow arrows represent glomerular swelling and deformation. Scale bars = 100 μm. **k** Renal human IgG deposit was evaluated by IF, and MFI was counted by Image J, scale bar = 200 μm. Data are representative of 3 (**a**–**c**) or 2 (**d**–**k**) independent experiments

SLE patients exhibit by B and T cell hyperactivity.^33,34^ To investigate the effect of 2D4 on B and T cells in SLE individuals, the PBMCs of SLE donors were cultured ex vivo. Emphasizing the pivotal role of excessive autoantibody production in SLE pathology, we first assessed 2D4’s effect on antibody production by PBMCs. Exposing PBMCs from SLE donors to 2D4 significantly inhibited the secretion of IgG1, IgG2, IgG3, and IGA, without lowering IgM and IgD (Fig. 7a–b). As both 2D4 and Fc are humanized IgG4 isotype, this system could not evaluate the effect of 2D4 on IgG4. To assess the impact of 2D4 on SLE T cells, we treated patients’ PBMCs with anti-CD3/28 beads, adding either 2D4 or Fc control. The addition of 2D4 significantly suppressed the release of IFN-γ, TNF-a, IL-17A and IL-5 by PBMCs from SLE patients (Fig. 7c). 2D4 did not affect cell viability in the short-term culture system and PBMCs was able to secret γc cytokines with or without anti-CD3/28 stimulation, such IL-21 (Supplementary Fig. 5a, b). Thus we hypothesize that 2D4 inhibits the secretion of inflammatory factors or antibodies by blocking the action of endogenous γc cytokines from PBMCs.

To better elucidate the therapeutic effects of 2D4 in SLE patients, we transferred PBMCs from SLE patients to NOD-SCID mice to induce a humanized lupus-like mouse model (GVHD).^35,36^ The clinical and laboratory characteristics of SLE patients are presented in supplementary Table 1. On the 18th day post-transferring SLE PBMCs, most NOD-SCID mice displayed the presence of human T cells and B cells in their blood (Supplementary Fig. 5c), which were then randomly assigned to two groups and given either 2D4 or Fc control treatment according to the bodyweight (three doses, one dose administered every three days) (Fig. 7d and Supplementary Fig. 5d). After 30 days of transfer, the Fc group showed significantly elevated levels of human-derived inflammatory factors such as IFN-γ and TNF-a in serum, which were almost completely inhibited by 2D4 (Fig. 7e, f). Elevated anti-dsDNA-IgG, characteristic of SLE disease, was detected in the plasma of each mouse in the Fc group but not in the mouse of the 2D4 group at the end-stage (Fig. 7g). The end-stage renal function of the mice was evaluated by measuring the urinary Alb/cre. This ratio was significantly lower in the 2D4 group compared to the Fc group (Fig. 7h). We then harvested the mice, detected inflammatory infiltrates in the kidneys, and conducted a thorough pathological evaluation of the renal lesions. As expected, expression of human *CD132*, *CD3*, *CD19*, *CD4* and *CD8* (which measures immune cell, T cell, B cell, Th cell, and CTL cell infiltration respectively) in whole renal tissues was reduced by 2D4 treatment (Fig. 7i and Supplementary Fig. 5e). Consistently, the Fc group exhibited caseous changes, including glomerular swelling and deformation (indicated by yellow arrows) as well as infiltration of inflammatory cells (indicated by green arrows). On the other hand, none of these pathological changes were observed in the mice from the 2D4 and Blank groups (Fig. 7j). In addition, deposition of human-driven antibodies (IgG) was observed in every mouse from the Fc group, but in only half of the mice in the 2D4 group and none in the Blank group (Fig. 7k). In conclusion, 2D4 protects against disease pathology and renal damage in humanized lupus-like mouse model (GVHD) by inhibiting the activity and function of T and B cells from SLE patients.

## Discussion

SLE patients, especially those with nephritis involvement, face significant unmet needs in treatment options. The formation of immune complexes due to autoantibodies produced by B cells is a crucial factor in SLE development. Current treatment strategies for SLE are mainly B-cell-centered, aiming to disrupt B-cell activation and survival. Despite success in certain autoimmune disease contexts, B cell–depleting agents like Rituximab, Ocrelizumab, Obinutuzumab and Obexelimab have exhibited favorable clinical effects in specific autoimmune disease settings but failed in clinical trials of SLE. For example, Rituximab failed to demonstrate effectiveness in lineage-based trials of SLE and LN.^37,38^ Although Belimumab, approved by the FDA in 2011, marked a significant milestone as the first new drug for SLE in over 50 years, it did not achieve disease improvement and flare reduction endpoints in 40–50% of SLE patients during trials.^39^ The intricate pathogenesis of SLE involves not only B-cell disorders but also central roles for T-cell over proliferation and activation. However, therapies successful in targeting T cells in various autoimmune diseases, such as Ustekinumab in psoriasis and inflammatory bowel disease, have not translated into success in phase III clinical trials for SLE.^40^ These challenges underscore the complexity of SLE compared to other autoimmune diseases. Targeting either B cells or T cells in isolation alone may not be sufficient, emphasizing the potential of combination therapies to offer more promising outcomes for the management of SLE.

In this study, we focused on CD132, which is highly expressed in both T and B cells. We observed that CD132 was significantly upregulated in lymphocytes from SLE patients indicative of its pro-inflammatory role. Subsequently, we developed a humanized monoclonal anti-CD132 for SLE treatment. Considering that binding to different sites of CD132 may result in different blocking effects on the six upstream γc cytokines, we strategically designed 2D4 to minimize inhibition of Tregs, crucial for SLE protection. Notably, 2D4 demonstrates enhanced blocking of IL-21 while being less potent in inhibiting IL-2, thus avoiding disruption to the Treg cell proportion in vivo. Our experiments in pristane-induced SLE mouse model and PBMCs-induced humanized lupus-like mouse model (GVHD) consistently revealed the promising therapeutic efficacy of 2D4, aligning the activities and functions of both T and B cells. Furthermore, we compared 2D4 with Belimumab, which has been well-established in the treatment of SLE. We found that 2D4 exhibited superior effectiveness over Belimumab in protecting SLE mice against renal damage, underscoring its potential as a more robust therapeutic option for SLE.

JAK inhibitors, such as Tofacitinib, also target multiple immune cells to treat autoimmune diseases by interfering with multiple inflammatory factors. JAK inhibitors have been successful in many refractory autoimmune diseases, such as ulcerative colitis, rheumatoid arthritis, etc.^41,42^ However, the first generation of JAK inhibitors, which indiscriminately target multiple JAKs, affects more than 25 cytokines and growth factors at the same time, inevitably leading to a variety of side effects, including the risk of infection, venous thromboembolism, and major adverse cardiovascular events, prompting an FDA warning in 2021.^43^ The evolution to second-generation JAK inhibitors, with selectivity for JAK1, JAK2, or JAK3, such as Upadacitinib for JAK1, Baricitinib for JAK1/2, and Ritlecitinib for JAK3/TEC, has shown improved efficacy and a more favorable safety profile in specific autoimmune diseases. Ritlecitinib mainly targets JAK3, a direct downstream molecule of CD132, and has shown promising results in autoimmune diseases such as IBD, pemphigus, and vitiligo,^44–46^ indicating potential applications for 2D4. Despite similarities in targeting γc cytokines, Ritlecitinib’s safety profile aligns partially with that of 2D4. However, it broadly inhibits all γc cytokines, potentially affecting the proportion of Treg cells in the body. Besides, in patients with alopecia areata, Ritlecitinib led to dose-dependent reductions in T cells and NK cells without affecting CD19^+^ B cells,^46^ potentially compromising its efficacy in autoantibody-dominated autoimmune diseases. Although there are no documented reports of JAK3 inhibitor-based therapy for SLE, we posit that 2D4 may exhibit greater efficacy in autoantibody-mediated autoimmune disease than JAK3 inhibitors.

The limitations of these studies relate to the inherent caveats associated with animal models of human disease. Mouse models of lupus do not fully recapitulate disease pathobiology in humans, raising the possibility that the efficacy observed with 2D4 in the lupus models used in the present report may not precisely translate to human patients. To enhance the relevance of our findings, we not only utilized the pristane-induced SLE model but also established a humanized lupus-like mouse model (GVHD) by transferring PBMCs from patients with SLE into CD132-humanised mice. This approach aimed to maximize the confirmation of the efficacy of 2D4 in preclinical studies. While it is promising that the clinical validation of blocking γc cytokines by JAK3 inhibitor has been clinically validated in other autoimmune disorders such as IBD, pemphigus, and vitiligo.^44–46^ In our humanized lupus-like mouse model (GVHD), the detection of renal damage on day 28 revealed an accelerated disease progression marked by rapid weight loss and partial mouse mortality. Administering the drug at this stage proved challenging in reversing the disease state. Recognizing this, we initiated 2D4 treatment at day 18, acknowledging that this approach may not respond to patients already with severe renal injury. Future studies are planned to evaluate the impact of 2D4 on severe LN.

In summary, biologics targeting specific factors or pathogenic cell types in SLE have faced challenges in meeting the stringent criteria of clinical trials, with limited success in terms of efficacy. Given this scenario, our emphasis has shifted to CD132, a pivotal target involved in signaling multiple inflammatory factors and regulating a wide range of pathogenic cells in SLE. Our study is the first to investigate the therapeutic potential of targeting CD132 for SLE treatment, providing a novel avenue for therapeutic strategies in SLE. Moreover, our data strongly suggest that 2D4 is a promising candidate molecule for future clinical trials in the context of SLE.

## Materials and methods

### Study design

We conducted this study to investigate CD132’s role in SLE pathology and manipulate CD132 for SLE treatment. To block six γc cytokines differentially, we generated a comprehensive library of monoclonal antibodies using phage display, identified 2D4 through cascade screening. We conducted PK and pharmacodynamic studies of 2D4 in humanized mice and confirmed its efficacy in the pristane induced SLE mouse model and a humanized SLE model.

### Human

Peripheral blood or PBMCs of 58 individuals diagnosed with SLE at the Institute of Dermatology, Chinese Academy of Medical Sciences were used as the study subjects, and 30 healthy blood donors (HCs) were used as controls. PBMCs from another 20 HCs were used to screen a targeted monoclonal antibody and other experiments. Comprehensive information for the SLE individuals and corresponding HCs is available in Supplementary Table 1. Ethical approval was obtained from the Institute of Dermatology, Chinese Academy of Medical Sciences (No. 2021-KY-054 & 2019-KY-005) for studies involving human samples, and informed consent was obtained for all studies involving human samples.

### Mice

Female C57BL/6 J mice, NOD-SCID (*Prkdc*^*-/-*^ */ IL-2rg*^*-/-*^, Cat. No. 110586) mice and human FcRn transgenic mice (Cat. No. 110001) were purchased from Biocytogen Pharmaceuticals (Beijing, CN). BAFF/BAFFR humanized mice (BAFF^hu/hu^ /BAFFR ^hu/hu^) were produced by Biocytogen Pharmaceuticals (Cat. No. 111921), which were generated on a C57BL/6 J background. CD132^hu/hu^ mice were generated by gene targeting in ES cells on a C57BL/6 J background. The exons 1-8 of mouse *Cd132* gene that encode the full-length protein were replaced by human CD132 exons 1-8, as shown in supplementary Fig. 3a. All mice were housed in a temperature-controlled and humidity-controlled facility with free access to food and water, following a 12-hour dark/light cycle. Protocols were carried out in line with the animal ethical and care norms of the Institute of Dermatology, Chinese Academy of Medical Sciences (2022-DW-004) and approved by local government authorities to ensure compliance.

### Generation of 2D4 and REGN7257

2D4 was isolated from an antibody phage display and generated as a human IgG4 as described.^47^ In short, female Balb/C mice were subjected to subcutaneous injection with 50 µg of recombinant protein comprising the ectodomain of human CD132 (spanning from amino acid L23 to A262). At peak titers, all mice were harvested, and spleen cells were collected for the extraction of RNA sequences of antibody variable regions. M13 phage display technology was used according to standard protocols.^48^ The ectodomain of human CD132, our target antigen, was immobilized onto polystyrene tubes for three rounds of panning. Specific binders were subsequently identified via ELISA. These selected fragment antigen-binding (Fabs) were then recloned and produced in an IgG4 format customized for subsequent investigations. Finally, the humanization of the 2D4 was accomplished through complementary determining regions (CDR) grafting.^49^ REGN7257 was generated by Keymed Biosciences based on sequence ID nos. 357 and 359 from US patent application US 2020/015841, which was applied by Regeneron Pharmaceuticals.

### Mouse KLH immunization model

8-10-week-old CD132^hu/hu^ or wild type C57BL/6 J female mice were randomly assigned (*n* > 5 per group). All mice except the Blank group were immunized with 200 μL KLH (0.5 mg/mL) (Millipore Sigma) emulsified in CFA via subcutaneous injection on day 0 and 7. Mice received either Fc control or 2D4 subcutaneous injection molar matched to 20 mg/kg. Mice were harvested on day 21, and blood and lymph nodes were collected for serum anti-KLH immunoglobulin analysis and lymphocyte immunophenotyping.

### Lupus mouse model

For pristane-induced lupus mice, the mice were administered one i.p. injection of 500 µL saline (as a control) or pristane (Millipore Sigma). For the humanized lupus-like mouse model (GVHD), PBMCs from 3 SLE patients (Supplementary Table 1) were obtained by standard ficoll separation, washed in Hank’s solution, suspended in RPMI, supplemented with 10% fetal calf serum, and injected IV into the 7–10-week-old DKO (*BALB-Rag2*^*−/−*^ *IL-2Rgc*^*−/−*^) mice at total of 1 × 10^7^ cells/mouse. To detect PBMC colonization, 50 μL of blood was collected in EDTA tubes every 7-10 days after PBMC inoculation. Red blood cells were lysed, and the PBMC was stained with various anti-human cell markers CD45, CD3, CD4, CD8, and CD19, and analyzed by flow cytometry.

### Flow cytometry

PBMCs from donors or mice or cell suspensions obtained from mouse lymph nodes through mechanical disruption and filtration were stained on ice in staining media (PBS with 2% FBS and 0.02% sodium Azide) with antibodies listed in Supplementary Table 2. The cells were washed and stained with the fixable viability dye zombie NIR (BioLegend) in PBS for 30 min, then were treated with mouse Fc-R block (BD Pharmingen, 553141) for 15 min at room temperature. Subsequently, surface staining was performed using specific marker antibodies for 45 min at 4 °C without light. To enable intracellular staining, the cells were fixed and permeabilized using the Transcription Factor Buffer Set (BD Pharmingen, 562574) and stained with fluorescent antibodies for 45 min at 4 °C. Flow cytometry analysis was conducted using FlowJo software version 10.8.1.

### Analysis of anti-KLH and anti-dsDNA antibody levels by ELISA

Antibody titers of KLH–specific and anti-dsDNA immunoglobulins in serum were measured with ELISA. For KLH-specific antibody detection, wells were coated with 10 μg/mL KLH (Solarbio) overnight at 4 °C. For mouse anti-dsDNA antibody analysis, wells were coated with 100 μg/mL dsDNA (Invitrogen) overnight at 4 °C. Wells were blocked with 10% BSA, and diluted serum was incubated in the wells for 1 hour at room temperature. HRP-labeled Fc-specific anti–mouse IgG1, IgG2c, IgG2b, IgG3, or IgM detection Ab was used, as listed in Supplementary Table 2. For human anti-dsDNA antibody analysis, wells were coated with 100 μg/mL dsDNA (Invitrogen) overnight at 4 °C. HRP-labeled Fc-specific anti–human IgG was used, as listed in Supplementary Table 2. Color development was with a 3,3′,5,5′-tetramethylbenzidine Substrate Kit (MCE) and analyzed on the Synergy H1 microplate reader (BioTek). Absorbance at 450 nM and 570 nM was used to measure anti-KLH and anti-dsDNA antibodies; there was no standard curve for the assay. The data of each chart was collected on the same plate at the same time to compare OD450-OD570 values.

### Quantitative RT-PCR assay

Total RNA was extracted from mouse kidneys using Trizol reagent (Thermo Fisher Scientific). The RNA quality and concentration were determined using a NanoDrop spectrophotometer (Thermo, ND-2000). cDNA was synthesized from the extracted RNA using the High-Capacity RT kit (RR036A, TaKaRa, Tokyo, Japan). The cDNA was diluted to a final volume of 200 µL. 4 µL diluted cDNA was used for each qPCR containing SYBR Green Premix Ex Taq II (RR820A, TaKaRa). Relative expression of genes was quantitated by the SYBR Green dye-based assay (Applied Biosystems) using GAPDH RNA as the housekeeping control. The gene mRNA levels were normalized to the control group and calculated using the formula 2^-ΔΔCt^ (where ΔΔCt = ΔCt of experimental group - ΔCt of control group). The primer sequences were listed in supplementary Table 3.

### Cell stimulation

CD19^+^ B cells and CD4^+^ T cells are extracted from the fresh peripheral blood of healthy donors by negative magnetic selection (BioLegend, Cat. No. 480082 & 480130). Purified B and T cells were cultured at 0.5 million cells/well in 96-well plates in 100 uL complete medium, incubated with IgG4 isotype (Fc control) or REGN7257 at 30 µg/mL concentration. B cells were cultured for 2-3 days with the stimulation of RB48 at a final concentration of 1 µg/mL, and cell supernatants were harvested for further immunoglobulins analysis. For T cell culture, anti-CD3 antibodies (BioLegend, Cat. No. 300438; 5 µg/mL) were precoated in a 96-well plate at 4 °C overnight, CD4^+^ T cells were plated into the medium with anti-CD28 antibodies (BD, Cat. No. 555725; 1 µg/mL) for 2-4 days, and cell supernatants were harvested for the further cytokines analysis.

PBMCs were isolated by density gradient centrifugation using Ficoll-Paque (GE Healthcare). PBMCs from SLE patients were cultured at 0.5-1 million cells/well in 96-well plates for 3-5 days in complete medium with or without the stimulation of anti-CD3/CD28 Dynabeads (Invitrogen, Cat. No. 11161D), incubated with Fc control or REGN7257 at concentration 30 μg/mL. Cell supernatants were harvested for further immunoglobulins and cytokines analysis.

The complete medium was RPMI 1640 medium (Gibco) supplemented with 10% FBS (Gibco), 1% non-essential amino acids (Gibco), 1 mM sodium pyruvate 33 (Gibco), 2mM L-glutamine (Gibco), 500 U/mL Penicillin and Streptomycin (Gibco).

### Cytokines and immunoglobulins analysis by Cytometric Bead Array

Different isotypes of human immunoglobulins, mouse cytokines and human cytokines in cell culture and mouse blood were quantitatived by the following LEGENDplex™ kits from BioLegend, Human Ig Isotyping Panel (8-plex, IgG1, IgG2, IgG3, IgG4, IgE, IgA, IgM, IgD) (Cat. No. 740637), HU Th Cytokine Panel (12-plex, IL-5, IL-13, IL-2, IL-6, IL-9, IL-10, IFN-γ, TNF-α, IL-17A, IL-17F, IL-4, IL-22) (Cat. No. 741028) and Mouse Th Cytokine Panel (12-plex, IFN-γ, IL-5, TNF-α, IL-2, IL-6, IL-4, IL-10, IL-9, IL-17A, IL-17F, IL-22, IL-13) (Cat. No. 741043). LEGENDplex™ kits were used according to the manufacturer’s protocols. Twenty-five microliters of cell supernatant or diluted serum were incubated with 25 μL incubation buffer and 25 μL bead mix per well for 2 h at room temperature at 800 rpm on a short-radius platform shaker. The beads were centrifuged at 300×g for 5 min, resuspended in 150 μL wash buffer supplied with the kit, and centrifuged again. The washed beads were stained with 25 μL detection antibody mix for 1 h, followed by the addition of 25 μL of PE-streptavidin mix and incubated for an additional 30 min. The beads were washed once more, as above. Standard curves for each cytokine were performed by staining beads with dilutions of the Legendplex kit-supplied standard mix. Beads were analyzed on a Cytoflex flow cytometer, and flow assay results were imported into and determined through the LEGENDplex software.

### Anti-CD132 in vitro activity evaluation systems

#### Phosflow assays

Human PBMCs were isolated from fresh whole blood using a density gradient centrifugation method. Briefly, human whole blood was diluted with an equal volume of PBS and layered onto Ficoll-Paque in a new conical tube, followed by centrifugation. The PBMC-containing layer was carefully extracted and washed twice with PBS. Subsequently, the cells were resuspended in RPMI 1640 medium (BasalMedia) and dispensed into 96-well plates. Serial dilutions of 2D4, REGN7257, or isotype control were suspended in prewarmed 1640 medium and added to the cells and incubated at 37 °C for 30 min. Then fixed concentrations of cytokines (human IL-2 from SI HUAN SHEN WU bio-company, human IL-21 and IL-7 from Novoprotein, and human IL-15 from Acrobiosystems) was added to facilitate STAT protein phosphorylation. The stimulation was halted by adding warm Fix buffer I (BD Biosciences) into each well, followed by a 10-minute incubation at 37 °C. Cells were then washed twice with wash buffer (PBS containing 1% v/v fetal calf serum (FBS, excell bio)) and permeabilized by cold Perm Buffer III (BD Biosciences). Cells were incubated at 4 °C for 30 min and washed with wash buffer. Permeabilized human PBMCs were stained at 4 °C for 45 min with Immunophenotyping reagent combinations to analyze STAT phosphorylation in CD3^+^ T cells (CD3-PE-Ab, and phospho–STAT-AF647 Ab (BD Biosciences) diluted in PBS) and then washed twice with wash buffer. Data were acquired on a BD FACSCelesta™ Flow Cytometer using the high-throughput sampler (HTS) attachment (BD Biosciences).

### NK92 cell IFN-γ release assay

Human NK92 cells (Procell) were maintained in continuous culture in MEM Alpha Medium (BasalMedia) supplemented with 12.5% horse serum (Procell), 12.5% FBS, 0.2 mM inositol, 0.02 mM folic acid, 0.1 mM 2-mercaptoethanol, antibiotics, and 200 IU/mL human IL-2. In all the experiments, NK92 cells were removed from the IL-2-containing medium 16 h before stimulation. Serial dilutions of 2D4, REGN7257 or Fc control were added in continuous culture without IL-2 to suspend NK92 cells and incubated at 37 °C for 30 min. Then cytokines were added and incubated for 72 h (IL-21) or 48 h (IL-2 and IL-15). Supernatants from cells were analyzed for IFN-γ production by ELISA using paired antibodies specific to IFN-γ (BD Biosciences) following the manufacturer’s instructions.

### M07E cell proliferation assay

M07E cells (ATCC) were maintained in RPMI 1640 plus 20% FBS and 10 ng/mL GM-CSF (Novoprotein). After overnight GM-CSF withdrawal in RPMI 1640 medium with 20% FBS, M07e cells were plated at 10^5^/mL in 96-well plates. Serial dilutions of 2D4, REGN7257 or Fc control were added to cells and incubated at 37 °C for 30 min, followed by stimulation with IL-9 and incubation for 120 h. Cell proliferation was measured using the CellTiter-Glo®Luminescent Cell Viability Assay.

### CD23 expression by human B cells assay

Human PBMCs were obtained using the method outlined in the Phosflow assays section. PBMCs were plated at 10^6^ cells/mL in 96-well plates with RPMI medium containing 10% FBS, 20 μm 2-mercaptoethanol, and antibiotics. Serial dilutions of 2D4, REGN7257, CM310 or Fc control were added to cells and incubated at 37 °C for 30 min, followed by stimulation with IL-4 and incubation for 48 h. Then, cells were washed twice in PBS and stained with anti-huCD23-FITC (BD Biosciences) and anti-huCD20-APC (BD Biosciences) for 30 min at 4 °C. CD23 expression was analyzed by flow cytometry, with quantification based on the MFI within the CD19-positive cell population.

### Electron microscopy data acquisition and image processing

Cryo-EM data were collected with a 300 kV Titan Krios electron microscope (Thermo Fisher Scientific, USA) equipped with a Falcon4i direct electron detector (Thermo Fisher Scientific, USA) operating in a counting mode. All movies were automatically recorded using EPU at a magnification of 165 K, with a physical pixel size of 0.722 Å and a total dose of 60 e- / Å2. 9,885 movies were collected with a defocus range from 1.0 µm to 2.0 µm and the slit width of the Thermo Scientific™ Selectris (Thermo Fisher Scientific, USA) was set to be 10 eV.

All image processing was performed using cryoSPARC v4.5.3 and Relion5. The movies were motion-corrected using path motion with cryoSPARC’s own implementation. Patch CTF estimation, 2D classification, heterogeneous refinement and Non-uniform Refinement were all performed in cryoSPARC. A total of 6,019,841 particles were auto-picked using Blob picking and extracted with 2×2 binning (192-pixel box size, 1.444 Å per pixel). Two rounds of 2D classification were performed to select a data set of good particles. The remaining 2,219,959 particles were subjected to Ab initio reconstruction. Then these selected particles were subjected to Ab initio and Heterogeneous Refinement, using four volumes as starting models. One of those four volumes showed a relatively intact complex with a resolution of 3.70 Å and the model of CD132 from 8EPA could be well-fitted to the volume. The refined coordinates were used for re-centering and re-extraction of particles using a box size of 384 pixels, with a pixel size of 0.722 Å/pixel. These particles were subjected to further refinement and the final density map was at 2.70 Å resolution. Local Resolution distribution of the refined map was estimated by Relion5.

### Statistical analysis

GraphPad Prism 9.5.0 was used for statistical analysis. The results are provided as mean ± SEM, with a normal distribution and equal variation between groups. Two-tailed unpaired Student’s *t*-tests were used to compare the two groups. One-way analysis of variance (ANOVA) was used to compare groups, followed by appropriate post hoc tests. When data had unequal variances or were not generally distributed between two groups, the 2-tailed Mann-Whitney U test was employed. Sample sizes were not predetermined using a statistical method, and mice were randomly assigned to different groups. To reduce potential bias, the researchers were blinded to the group assignment during analysis.

## Supplementary information

Supplementary figures

## Data Availability

All data supporting this paper are present within the paper and the Supplementary Materials. EMdata of 2D4 in complex with CD132 have been deposited under the following PDB accession numbers: PDB code 9JQT and EMDB code EMD-61741. The original data sets are also available from the corresponding author upon request. Single-cell transcriptome data of healthy and SLE donors analysed in Fig. 1g-i were from the National Bioscience Database Center Human Database with the accession number of E-GEAD-397 and JGAS000220.
