# Supplementary material for: 2D4, a humanized monoclonal antibody targeting CD132, is a promising treatment for systemic lupus erythematosus
Source: Signal Transduct Target Ther. 2024 Nov 17;9:323. doi: 10.1038/s41392-024-02017-6 (PMC11570697; doi:10.1038/s41392-024-02017-6)
Supplement: Supplementary file 1 — Supplementary figures [file 41392_2024_2017_MOESM1_ESM.docx]

**Supplementary Materials for**

**2D4, a humanized monoclonal antibody targeting CD132, is a promising treatment for systemic lupus erythematosus**

Huiqi Yin^1,2*^, Liming Li^1,2*^, Xiwei Feng^1,2*^, Zijun Wang^3^, Meiling Zheng^1,2^, Junpeng Zhao^1,2^, Xinyu Fan^1,2^, Wei Wu^1,2^, Lingyu Gao^1,2^, Yijing Zhan^1,2^, Ming Zhao^1,2#^, Qianjin Lu^1,2#^

^#^Correspondence to: Qianjin Lu ([qianlu5860@pumcderm.cams.cn](mailto:qianlu5860@pumcderm.cams.cn)) and Ming Zhao ([zhaoming301@pumcderm.cams.cn](mailto:zhaoming301@pumcderm.cams.cn))

**This PDF file includes:**

Figures. S1 to S5

Tables S1 to S3

Figure. S1.


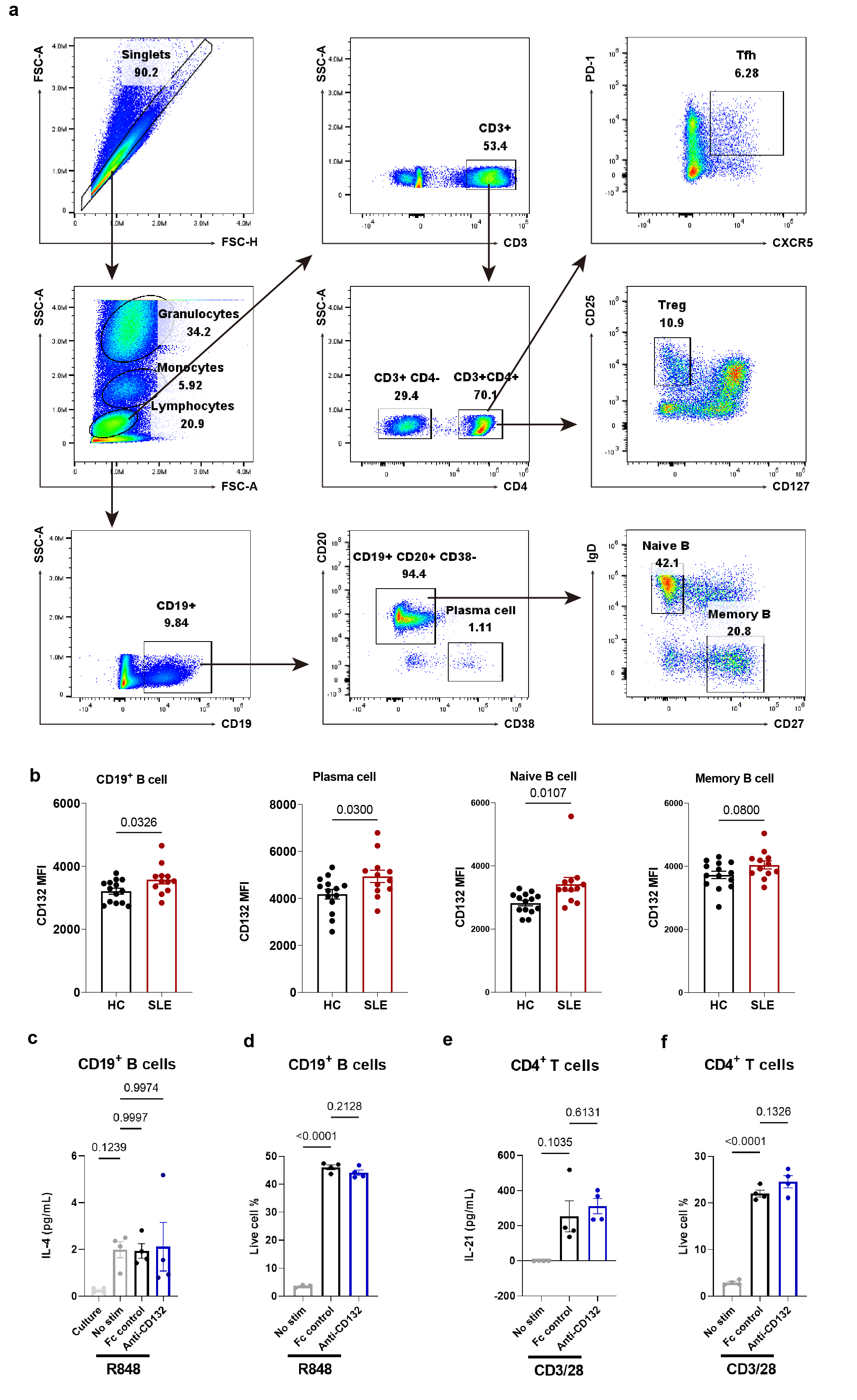


**Supplementary Figure 1.** Gating scheme for quantifying immune cell subsets in human PBMCs. Cells were gated away from debris in the FSC-A/SSC-A dot plot. Lymphocytes, monocytes, and granulocytes were gated from the FSC-H/FSC-A singlet gate, based on their cell size (FSC-A) and granularity (SSC-A). Lymphocytes were then analyzed by a CD3/SSC-A dot plot. The CD3^+^ cells were then analyzed by a CD4/SSC-A dot plot to identify CD4^+^ T cells and CD4^-^ T cells. CD4^+^ T cells were subsequently analyzed by a PD-1/CXCR5 dot plot to identify Tfh (PD-1^+^ CXCR5^+^) cells. CD4^+^ T cells were also analyzed by a CD25/CD127 dot plot to identify CD25^high^ CD127^-^ Treg cells. CD19^+^ B cells were gated from Lymphocytes with a CD19/SSC-A dot plot, which were subsequently analyzed by a CD220/CD38 dot plot to identify plasma cells (CD220^-^ CD38^+^). The CD220^+^ CD38^-^ cells were then analyzed by a IgD/CD27 dot plot to identify IgD^+^ CD27^-^ naive B cells and IgD^-^CD27^+^ memory B cells. FSC-A = forward scatter area; SSC-A = side scatter area; H = height; W = width. (b) Analysis of mean fluorescence intensity (MFI) of CD132 in B cell subtypes from HC and SLE (n>12). (c) IL-4 level in cell culture and supernatants of CD19^+^ B cells relative to Figure 1e, detected by ELISA assay. (d) Viability of CD19^+^ B cells relative to Figure 1e. (e) IL-21 level in cell supernatants of CD4^+^ T cells relative to Figure 1f, detected by ELISA assay. (f) Viability of CD4^+^ T cells relative to Figure 1f. In b, data were pooled from 2 independent experiments.

Figure. S2.


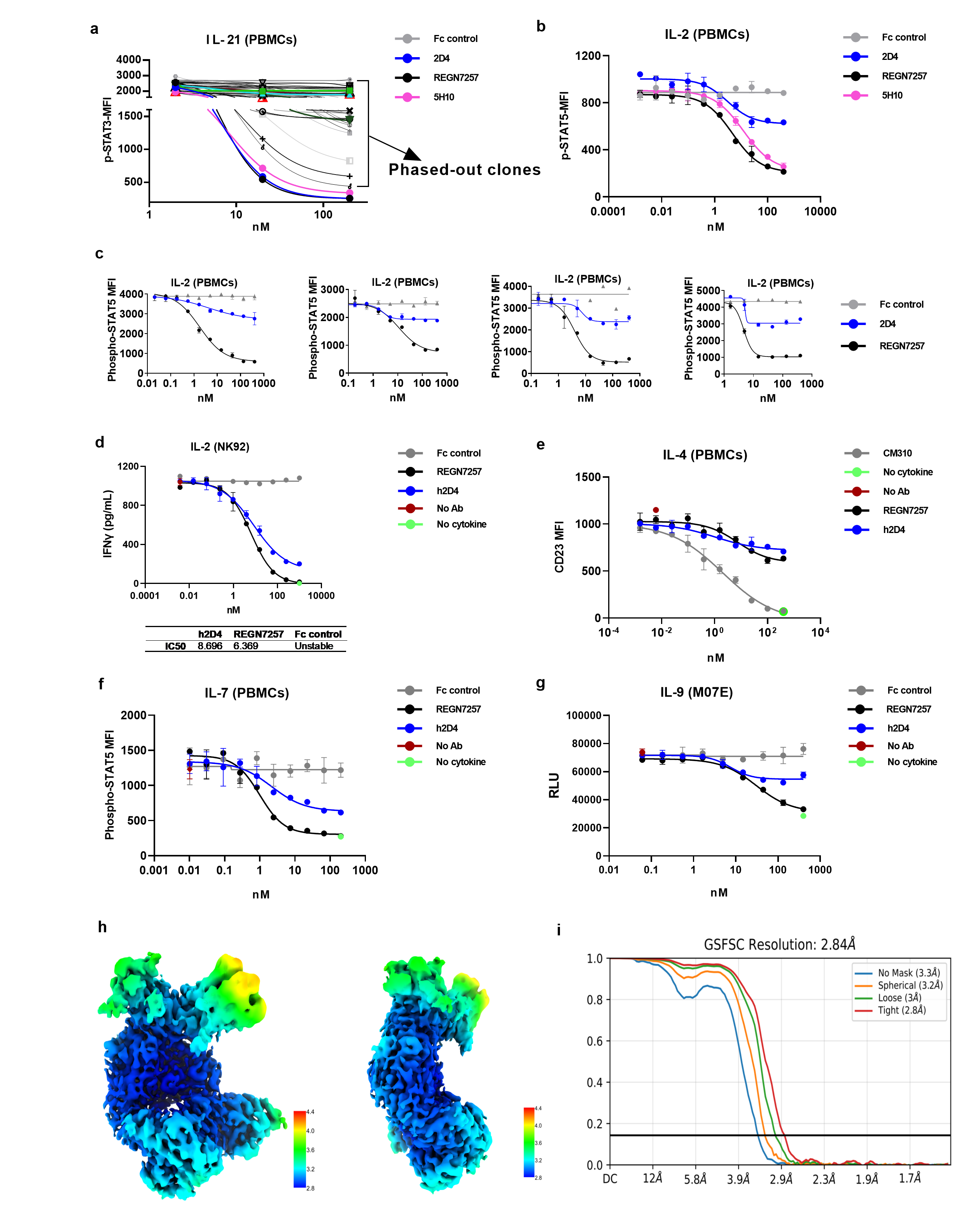


**Supplementary Figure 2.** Comparison of 2D4 and REGN7257 blocking activities in vitro. (a-c, f) Human PBMCs were blocked with gradient dilutions of 2D4, 5H10 or REGN7257 followed by stimulation with human IL-21, IL-2, and IL-7, inhibition of STATs phosphorylation (phospho-STAT3 or phospho-STAT5) in CD3^+^ T cells was assessed by flow cytometry. (d) NK92 cell lines were blocked with gradient dilutions of 2D4 or REGN7257 followed by stimulation with human IL-2, inhibition of IFNγ release was assessed by ELISA. (e) Human PBMCs were blocked with gradient dilutions of 2D4, REGN7257, or CM310 followed by stimulation with human IL-4, inhibition of CD23 expression in CD20^+^ B cells was assessed by flow cytometry. (g) M07E cells were blocked with gradient dilutions of 2D4 or REGN7257 followed by stimulation with human IL-9 and cell proliferation was measured using the CellTiter-Glo®Luminescent Cell Viability Assay. RLU = relative luminescence units, Bar charts show the mean±SD. (h) Cryo-EM map of the CD132-2D4 complex colored by local resolution ranging from 2.8 to 4.4 Å. (i) Fourier shell correlation (FSC) curve of the final map. Resolution was estimated using the Gold-standard FSC=0.143 criterion. In d-g, data are representative of 3 independent experiments.

Figure. S3.


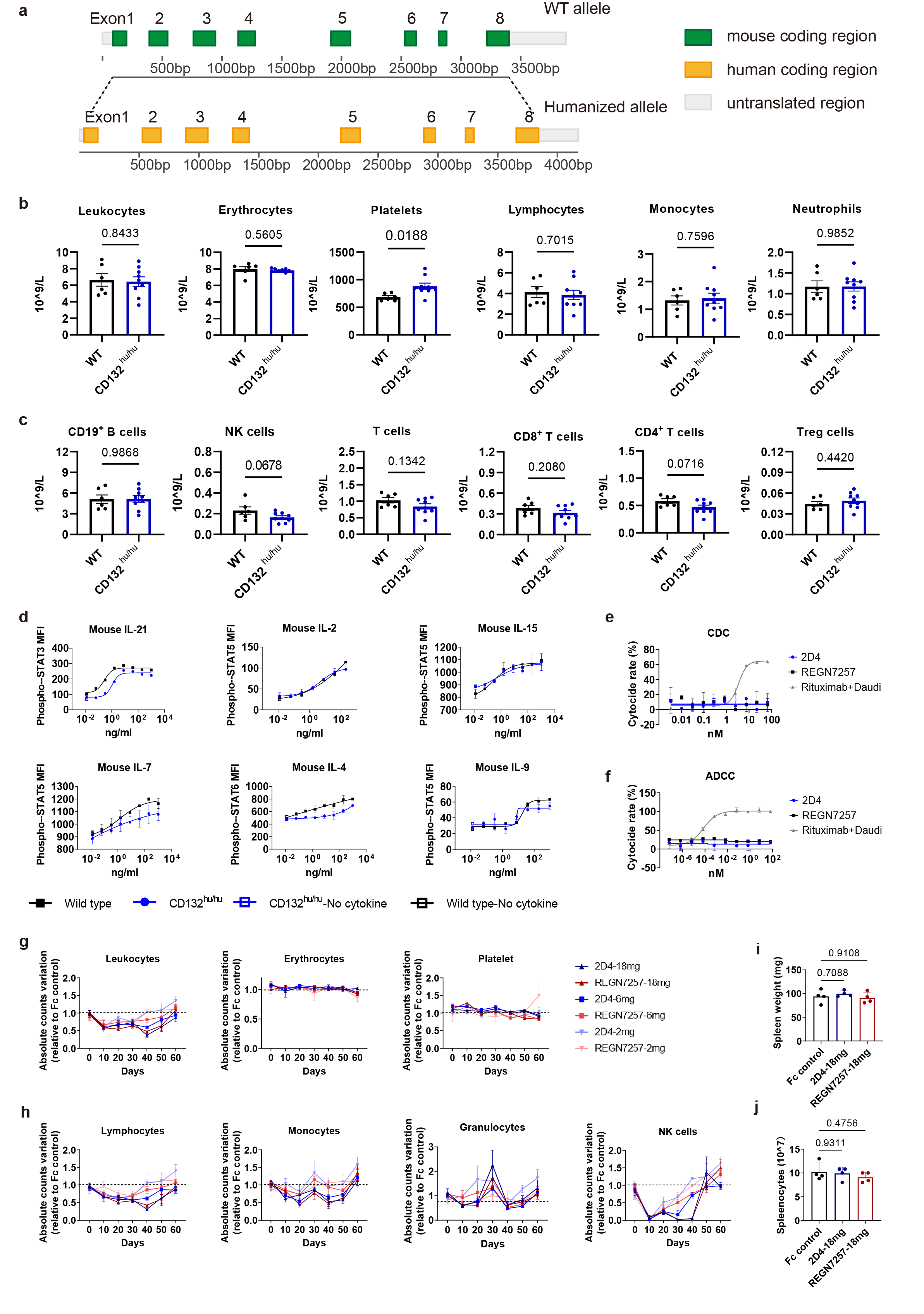


**Supplementary Figure 3.** Analysis of cell populations in peripheral blood and functional characterization of CD132^hu/hu^ mice (a) CD132^hu/hu^ mice were generated by replacing the mouse *Cd132* DNA sequence with its corresponding human sequence. (b) Hematology analysis of leukocyte, erythrocyte, platelet, lymphocyte, monocyte and neutrophils counts in the blood of CD132^hu/hu^ mice or WT mice (n>6). (c) Flow cytometric analysis of the proportion of B cell, NK, T cell, CD8^+^ T, CD4^+^ T and Treg cell in blood of CD132^hu/hu^ mice or WT mice (n>6). (d) Ex vivo analysis of STAT phosphorylation in mouse splenocytes from WT or CD132^hu/hu^ mice treated with increasing concentrations of mouse IL-21, IL-2, IL-15, IL-7, IL-4, or IL-9 for 15min. STAT phosphorylation in CD4^+^ T cells was assessed by flow cytometry. Error bars represent SD (n=3). (e) CDC, HEK293/CD132 target cells (HEK293 cells from ATCC engineered to express full-length CD132(AA M1 to T369) and Daudi target cells expressing human CD20 were incubated with 10% normal human serum (Schbio Biotechology) and a range of concentrations of 2D4 and REGN7257 (0.6fM to 70nM), or a CD20 IgG1 positive control Ab (Rituximab, 0.6fM to 70nM) for 4 hours. (f) ADCC, HEK293/CD132 target cells and Daudi target cells expressing human CD20 were incubated with human NK cells (4:1 ratio of effector to target cells) and a range of concentrations of 2D4 and REGN7257 (0.6fM to 70nM), or a CD20 IgG1 positive control Ab (Rituximab, 0.6fM to 70nM) for 4 hours. Cytotoxicity was determined using CellTiter-Glo®Luminescent Cell Viability Assay. (g-h) Absolute counts of leukocyte, erythrocyte, platelet, lymphocyte, monocyte, granulocyte counts and NK cells in blood were analyzed over time by hematology analysis and flow cytometric, changes in the cell counts of the experimental group relative to the Fc control group over time were shown (n>4). (i-j) Spleen weight and splenocytes counts at day 40 of high-dose group mice (n=4). MFI, mean fluorescence intensity. Data are representative of 2 (e-j) or 3 (b-d) independent experiments.

Figure. S4.


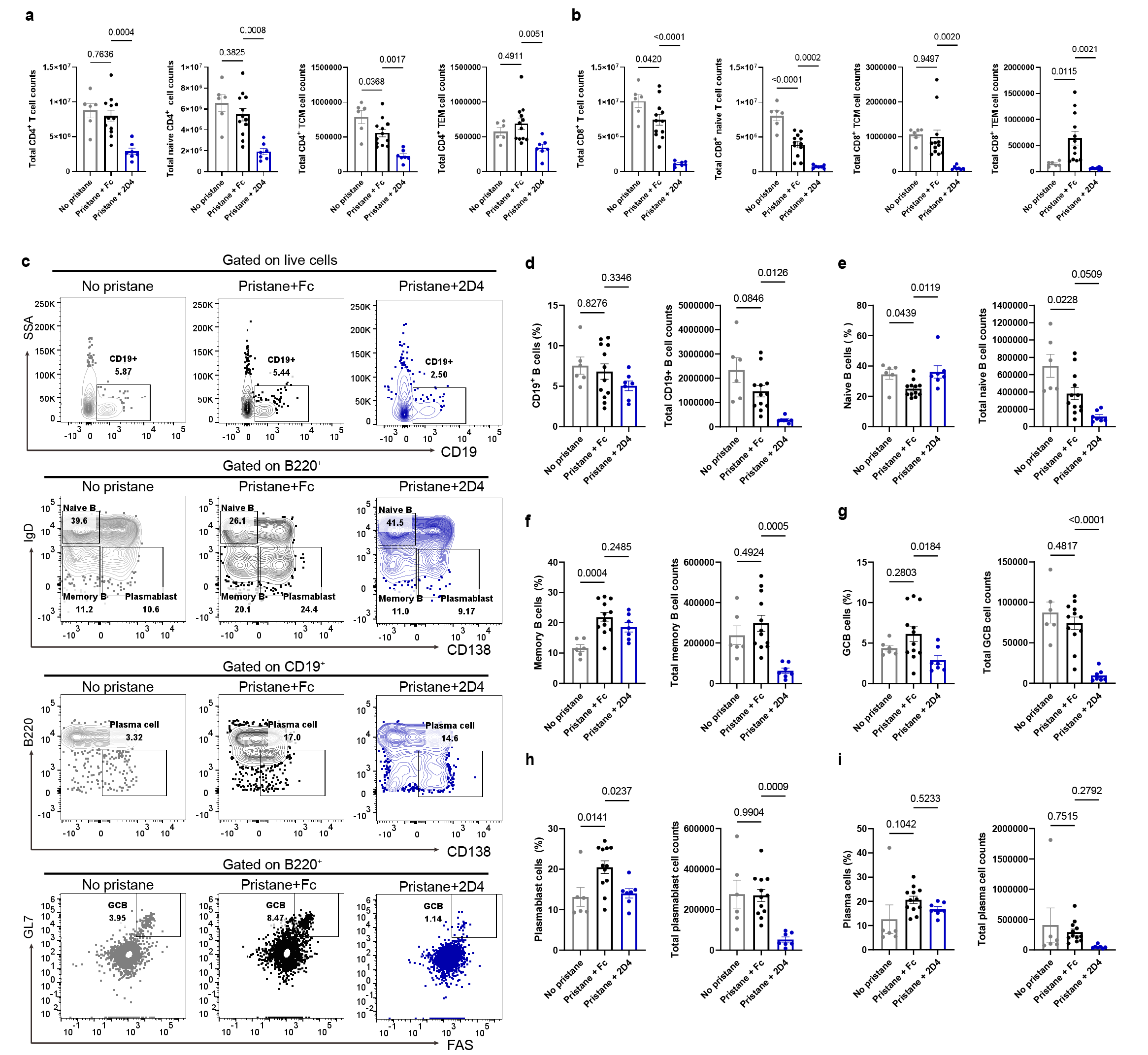


**Supplementary Figure 4.** Characterization of T and B cells in the draining lymph nodes (dLNs) of 2D4 treated mice (n>6). *Cd132^hu/hu^* mice were challenged with pristane on days 0 and received 20 mg/kg 2D4 or Fc control. At the end of treatment, dLNs were harvested, weighed, and characterized by flow cytometry. Populations of CD4^+^ T cell subsets (a) and CD8^+^ T cell subsets (b) in dLNs are presented. (c) Representative flow cytometry diagrams of B cell subtypes. (d-i) Percentage and population of CD19^+^ B cells (d), naive B cells (e), memory B cells (f), GC B cells (g), plasmablasts (h) and plasma cells (i) in dLNs. Symbols represent individual mice. Lines with whiskers show the mean ± SEM.

Figure. S5.

**
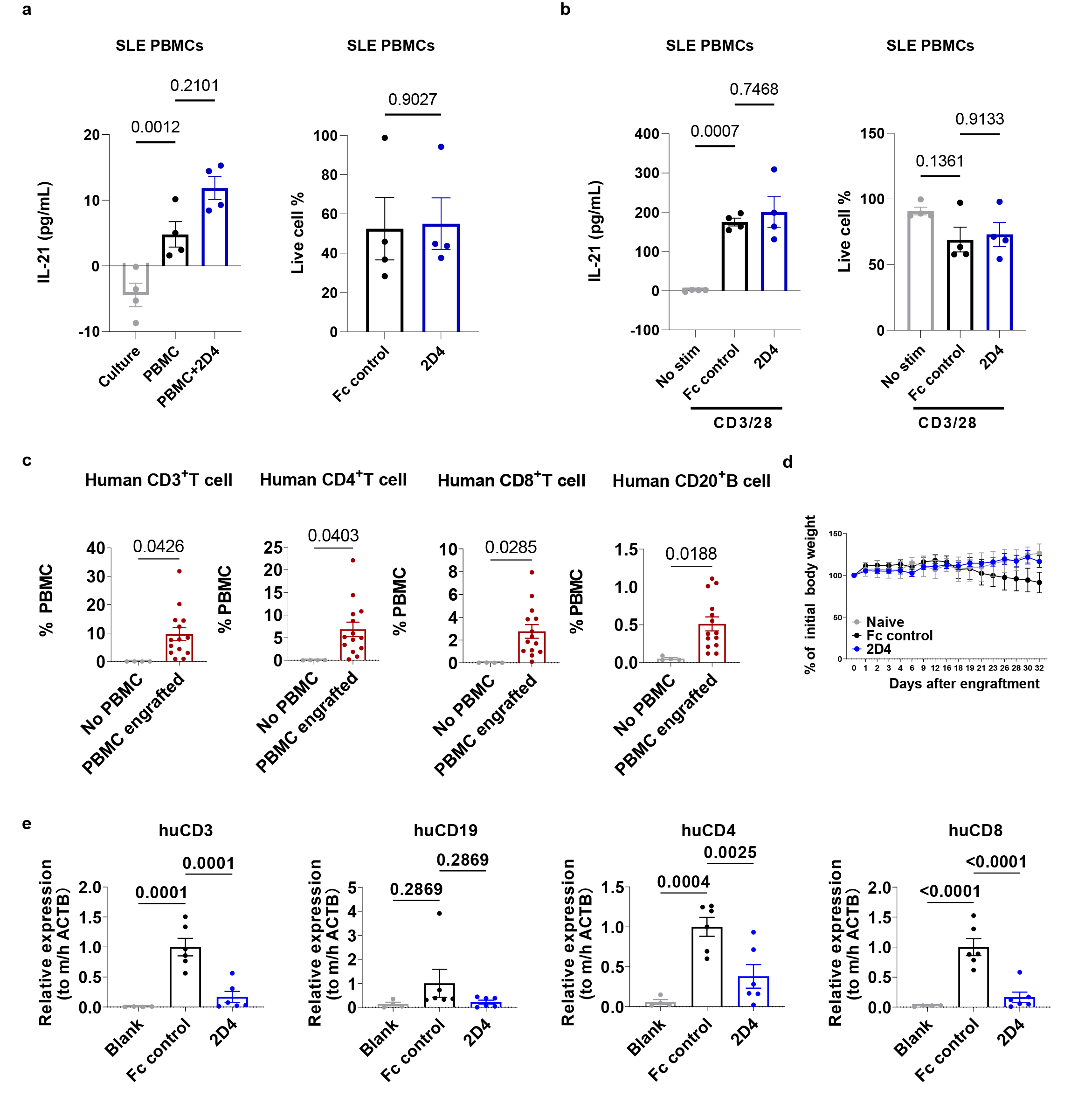
Supplementary Figure 5.** (a) IL-21 level in cell supernatants of SLE PBMCs and cell viability in the short-term culture system (n=4). (b) IL-21 level in cell supernatants of SLE PBMCs and cell viability with or without CD3/28 beads stimulation in the short-term culture system (n=4). Assessment of lymphocyte colonization in humanized lupus-like mice (GVHD) at day 18 post engraftment (c) and weight change over time (d) (n>4). (e) Renal tissue mRNA expression of human CD3, CD19, CD4 and CD8 were measured by real-time qPCR, expression versus mouse/human β-actin (ACTB) (n>4). Data are representative of 2 independent experiments.

**Supplementary Table 1. Information for SLE patients and Healthy control in related Figures**

| **Information of SLE and Heathy control in Figure 1a** | | | | | | | | |
| --- | --- | --- | --- | --- | --- | --- | --- | --- |
| SLE | | | | | | | Healthy control | |
| Age Gender | SLEDAI | Anti-dsDNA (IU/mL) | ANA | C3 (g/L) | Proteinuria | Medications | Age | Gender |
| 48 F | 5 | 44.6 | 0.486111111 | + | - | Hydroxychloroquine 200mg/day | 37 | F |
| 27 M | 16 | + | 0.152777778 | + | - | Hydroxychloroquine 200mg/day, | 27 | M |
|  |  |  |  |  |  | Asaison 12mg/day | 25 | F |
| 47 M | 6 | >2 | 0.263888889 | - | - | Untreated | 23 | F |
| 34 M | 8 | 2 | 1:40 | - | - | Metacortandracin 15mg/day | 32 | F |
| 25 F | 6 | - | 1:40 | - | - | Hydroxychloroquine 300mg/day | 25 | F |
| 26 F | 12 | >300 | 0.486111111 | + | - | Metacortandracin 25mg/day, | 32 | M |
|  |  |  |  |  |  | Tacrolimus 2mg/day |  |  |
| 14 F | 4 | 134 | 0.486111111 | + | - | Metacortandracin 4mg/day, |  |  |
|  |  |  |  |  |  | Hydroxychloroquine 200mg/day |  |  |
| 37 F | 2 | 95.9 | 1:40 | - | - | Metacortandracin 10mg/day, |  |  |
| 26 F | 2 | ± | 1:40 |  | - | Metacortandracin 6mg/day, |  |  |
|  |  |  |  |  |  | Hydroxychloroquine 200mg/day |  |  |
| **Information of SLE and Heathy control in Figure 1b-c.** | | | | | | | | |
| SLE | | | | | | | Healthy control | |
| Age Gender | SLEDAI | Anti-dsDNA (IU/mL) | ANA | C3 (g/L) | Proteinuria | Medications | Age | Gender |
| 56 F | 1 | - | 1:40 | 0.88 | - | Methylprednisolone 4mg/day | 17 | M |
| 17 M | 2 | - | 0.263888889 | 1.43 | - | Methylprednisolone 5mg/day | 31 | M |
| 55 M | 1 | - | - | 1.14 | - | Untreated | 41 | F |
| 80 F | 4 | - | - | 1.4 | + | Untreated | 19 | F |
| 26 F | 2 | - | 0.263888889 | 0.9 | - | Methylprednisolone 4mg and | 33 | F |
|  |  |  |  |  |  | Hydroxychloroquine 200mg | 66 | F |
|  |  |  |  |  |  | every other day | 44 | F |
| 23 F | 2 | + | 0.486111111 | 0.51 | - | Untreated | 31 | M |
| 29 M | 0 | ± | 0.097222222 | 0.7 | - | Untreated | 23 | M |
| 24 M | 2 | - | 0.263888889 | 0.9 | 3+ | Methylprednisolone 7.5mg/day | 18 | F |
| 41 F | 2 | - | 0.097222222 | 0.63 | 3+ | Mycophenolate mofetil 1.5mg every other day | 19 | F |
| 39 F | 8 | - | 0.152777778 | 0.29 | - | Methylprednisolone 7.5mg/day, | 10 | F |
|  |  |  |  |  |  | Methotrexat 15mg/week, | 40 | F |
|  |  |  |  |  |  | Hydroxychloroquine 200mg/day | 36 | F |
| 39 M | 4 | - | 0.097222222 | 0.96 | + | Methylprednisolone 4mg/day, | 24 | F |
|  |  |  |  |  |  | Mycophenolate mofetil 4mg/day, | 31 | F |
| 51 M | 0 | - | 1:40 | 1.13 | - | Untreated | 30 | F |
| 34 M | 0 | - | 0.097222222 | 1.18 | - | Methylprednisolone 4mg/day, | 30 | F |
| 10 F | 0 | - | 0.486111111 | 1.13 | - | Untreated | 39 | F |
| 21 F | 10 | 2+ | 0.486111111 | 0.34 | 2+ | Methylprednisolone 10mg/day, |  |  |
| 12 F | 7 | ± | 0.152777778 | 0.77 | + | Methylprednisolone 20mg/day, |  |  |
| **Information of SLE and Heathy control in supplementary Figure 1b.** | | | | | | | | |
| SLE | | | | | | | Healthy control | |
| Age Gender | SLEDAI | Anti-dsDNA (IU/mL) | ANA | C3 (g/L) | Proteinuria | Medications | Age | Gender |
| 41 F | 2 | - | 0.097222222 | 0.63 | 3+ | Mycophenolate mofetil 1.5mg every other day | 18 | F |
| 39 F | 8 | - | 0.152777778 | 0.29 | - | Methylprednisolone 7.5mg/day, | 19 | F |
|  |  |  |  |  |  | Methotrexat 15mg/week, | 10 | F |
|  |  |  |  |  |  | Hydroxychloroquine 200mg/day | 40 | F |
| 39 M | 4 | - | 0.097222222 | 0.96 | + | Methylprednisolone 4mg/day, | 36 | F |
|  |  |  |  |  |  | Mycophenolate mofetil 4mg/day, | 24 | F |
| 51 M | 0 | - | 1:40 | 1.13 | - | Untreated | 31 | F |
| 34 M | 0 | - | 0.097222222 | 1.18 | - | Methylprednisolone 4mg/day, | 30 | F |
| 10 F | 0 | - | 0.486111111 | 1.13 | - | Untreated | 30 | F |
| 21 F | 10 | 2+ | 0.486111111 | 0.34 | 2+ | Methylprednisolone 10mg/day, | 39 | F |
| 12 F | 7 | ± | 0.152777778 | 0.77 | + | Methylprednisolone 20mg/day, | 38 | F |
| 44 F | 2 | - | 0.097222222 | 1.12 | - | Methylprednisolone 4mg/day, | 31 | F |
|  |  |  |  |  |  | Mycophenolate mofetil 750mg every other day | 26 | F |
| 19 M | 2 | - | 0.097222222 | 1 | - | Untreated | 12 | M |
| 30 F | 4 | - | 0.263888889 | 0.9 | - | Methylprednisolone 4mg/day |  |  |
| 31 F | 0 | - | 0.152777778 | 1.06 | + | Untreated |  |  |
| **Information of Untreated SLE in Figure 1d.** | | | | | | |  |  |
| Age Gender | SLEDAI | Anti-dsDNA (IU/mL) | ANA | C3 (g/L) | Proteinuria | Medications |  |  |
| 69M | 18 | + | 1:40 | 1.18 | 2+ | Untreated |  |  |
| 30F | 12 | +, >300 | 0.486111111 | 0.88 | - | Untreated |  |  |
| 58F | 7 | - | 1:40 | 0.94 | - | Untreated |  |  |
| 59F | 6 | +, 37.8 | 0.097222222 | 1.19 | + | Untreated |  |  |
| 34F | 4 | - | 0.486111111 | 0.92 | - | Untreated |  |  |
| 42F | 4 | - | 1:40 | 1.16 | + | Untreated |  |  |
| 29F | 3 | +, >200 | 2:20 | 0.84 | - | Untreated |  |  |
| 15F | 2 | - | 0.486111111 | 0.8 | / | Untreated |  |  |
| 30F | 2 | - | 0.097222222 | 0.59 | - | Untreated |  |  |
| 19M | 2 | - | 0.097222222 | 1 | -w | Untreated |  |  |
| 30F | 0 | - | 0.152777778 | 0.99 | - | Untreated |  |  |
| 20M | 0 | - | 1:40 | 1.04 | - | Untreated |  |  |
| **Information of SLE in Figure 7a-b.** | | | | | | |  |  |
| Age Gender | SLEDAI | Anti-dsDNA (IU/mL) | ANA | C3 (g/L) | Proteinuria | Medications |  |  |
| 28F | 0 | - | 1:40 | 0.76 | ± | Untreated |  |  |
| 11F | 0 | ± | 1:40 | 1.04 | - | Metacortandracin 2.5mg/day |  |  |
| 31F | 6 | ± | 0.486111111 | 0.72 | - | Metacortandracin 10mg/day, |  |  |
|  |  |  |  |  |  | Mycophenolate mofetil 1.5g/day |  |  |
| 34F | 0 | ± | 0.486111111 | 0.92 | - |  |  |  |
| **Information of SLE in Figure 7c.** | | | | | | |  |  |
| Age Gender | SLEDAI | Anti-dsDNA (IU/mL) | ANA | C3 (g/L) | Proteinuria | Medications |  |  |
| 39F | 0 | - | 1:40 | 1.46 | - | Untreated |  |  |
| 27F | 0 | - | 0.486111111 | 1.03 | - | Metacortandracin 4mg/day, |  |  |
|  |  |  |  |  |  | Hydroxychloroquine 200mg/day |  |  |
| 60M | 0 | - | 0.486111111 | 1.39 | - | Metacortandracin 10mg/day |  |  |
| 45F | 2 | - | 0.097222222 | 1.12 | - | Metacortandracin 4mg/day， |  |  |
|  |  |  |  |  |  | Mycophenolate mofetil 750mg/BID |  |  |
| 14F | 1 | ± | 0.263888889 | 1.18 | - | Metacortandracin 4mg/day， |  |  |
|  |  |  |  |  |  | Mycophenolate mofetil 750mg/BID |  |  |
| 34 F | 2 | - | 0.097222222 | 0.73 | ± | Untreated |  |  |
| 36 F | 0 | - | 0.152777778 | 0.89 | - | Methylprednisolone 7.5mg/day |  |  |
| 38F | 4 | - | 0.152777778 | 0.88 | - | Methylprednisolone 4mg/day |  |  |
| 47 F | 0 | - | 0.097222222 | 1.15 | - | Untreated |  |  |
| 48 F | 2 | - | 0.097222222 | 1 | - | Untreated |  |  |
| **Information of SLE in Figure 7d.** | | | | | | |  |  |
| Age Gender | SLEDAI | Anti-dsDNA (IU/mL) | ANA | C3 (g/L) | Proteinuria | Medications |  |  |
| 46 M | 0 | 10.5 | 1:40 | 1.1 | - | Methylprednisolone 4mg/day, |  |  |
|  |  |  |  |  |  | Mycophenolate mofetil 0.75mg/day |  |  |
| 65 M | 0 | <2 | 0.097222222 | 1.21 | - | Hydroxychloroquine 200mg/day |  |  |
| 58 M | 10 | >300 | 0.486111111 | 0.91 | + | Methylprednisolone 5mg/day, |  |  |
|  |  |  |  |  |  | Hydroxychloroquine 200mg/day, |  |  |
|  |  |  |  |  |  | Mycophenolate mofetil 0.75mg/day |  |  |

**Supplementary Table 2. Antibodies used in Flow Cytometry and Immunofluorescence Analyses**

| **Mouse** | | | |
| --- | --- | --- | --- |
| **Antibody** | **Brand** | **Catalog** | **Application** |
| FC block | Biolegend | 101302 | Flow cytometry |
| CD45.2 APC | Biolegend | 109814 | Flow cytometry |
| CD3 pc5.5 | BD | 560527 | Flow cytometry |
| CD4 AF700 | BD | 557956 | Flow cytometry |
| NK-1.1 BV650 | Biolegend | 108736 | Flow cytometry |
| CD8a APC-CY7 | BD | 557654 | Flow cytometry |
| CD25 BB515 | BD | 564424 | Flow cytometry |
| CD62L PE-CY7 | BD | 560516 | Flow cytometry |
| CD44 BV421 | BD | 563970 | Flow cytometry |
| CD19 BV510 | BD | 562956 | Flow cytometry |
| Foxp3 PE | BD | 563101 | Flow cytometry |
| CXCR5 | BD | 551960 | Flow cytometry |
| APC Streptavidin | Biolegend | 405207 | Flow cytometry |
| CD3 BV605 | Biolegend | 100237 | Flow cytometry |
| CD45R (B220) FITC | Biolegend | 103206 | Flow cytometry |
| PD-1 BB700 | BD | 566514 | Flow cytometry |
| Aqua BV510 | Biolegend | 423102 | Flow cytometry |
| CD19 APC-CY7 | BD | 557655 | Flow cytometry |
| IgD PerCP-Cy5.5 | Biolegend | 405710 | Flow cytometry |
| CD138 BV421 | Biolegend | 142508 | Flow cytometry |
| GL-7 AF647 | BD | 561529 | Flow cytometry |
| Fas(CD95) PE-CF594 | BD | 562499 | Flow cytometry |
| CD3 FITC | Biolegend | 100204 | Flow cytometry |
| CD25 BV786 | BD | 564023 | Flow cytometry |
| CD45R (B220) PE-CY7 | Biolegend | 103222 | Flow cytometry |
| HRP-Goat-Anti-Mouse IgG1 | Abclonal | AS066 | Immunofluorescence |
| HRP-Goat-Anti-Mouse IgG2c | SouthernBiotech | 1078-05 | Immunofluorescence |
| HRP-Goat-Anti-Mouse IgG2b | Abcam | Ab97250 | Immunofluorescence |
| HRP-Goat-Anti-Mouse IgG3 | Invitrogen | M32067 | Immunofluorescence |
| HRP-Goat-Anti-Mouse IgM | Proteintech | SA00012-6 | Immunofluorescence |
| **Human** | | | |
| **Antibody** | **Brand** | **Catalog** | **Application** |
| FC block | Biolegend | 422302 | Flow cytometry |
| CD3 AF700 | BD | 557917 | Flow cytometry |
| CD4 APC-CY7 | BD | 557871 | Flow cytometry |
| CD19 BV650 | BD | 563226 | Flow cytometry |
| CD25 BV421 | BD | 564033 | Flow cytometry |
| CD127 APC | Biolegend | 351316 | Flow cytometry |
| CD132 PE | Biolegend | 338606 | Flow cytometry |
| CXCR5 BV711 | Biolegend | 356934 | Flow cytometry |
| PD-1 PE-CY7 | BD | 561272 | Flow cytometry |
| CD38 PerCP-Cy5.5 | BD | 551400 | Flow cytometry |
| CD27 BV421 | BD | 562513 | Flow cytometry |
| CD69 PE-CY7 | BD | 557745 | Flow cytometry |
| HRP-Goat-Anti-Human IgG | Jakson | 109-035-098 | Immunofluorescence |

**Supplementary Table 3. Primers for qPCR**

| **Species** | **Gene** | **Direction** | **Sequence (5’→3’)** |
| --- | --- | --- | --- |
| Mouse | *Il1a* | Former | GCACCTTACACCTACCAGAGT |
|  |  | Reverse | AAACTTCTGCCTGACGAGCTT |
| Mouse | *Ifnα* | Former | ATGAACGCTACACACTGCATC |
|  |  | Reverse | CCATCCTTTTGCCAGTTCCTC |
| Mouse | *Ifnγ* | Former | ATGAACGCTACACACTGCATC |
|  |  | Reverse | CCATCCTTTTGCCAGTTCCTC |
| Mouse | *Gzmb* | Former | GCCCACAACATCAAAGAACAGG |
|  |  | Reverse | CCAACCAGCCACATAGCACAC |
| Mouse | *Tnfβ* | Former | CCACCTCTTGAGGGTGCTTG |
|  |  | Reverse | CATGTCGGAGAAAGGCACGAT |
| Mouse | *Il6* | Former | CTGCAAGAGACTTCCATCCAG |
|  |  | Reverse | AGTGGTATAGACAGGTCTGTTGG |
| Mouse | *Cd132* | Former | GTGCAGCCACTATCTATTCTCTG |
|  |  | Reverse | GTGAAGTGTTAGGTTCTCTGGAG |
| Mouse | *Cd3* | Former | ATGCGGTGGAACACTTTCTGG |
|  |  | Reverse | GCACGTCAACTCTACACTGGT |
| Mouse | *Cd4* | Former | TCCTAGCTGTCACTCAAGGGA |
|  |  | Reverse | TCAGAGAACTTCCAGGTGAAGA |
| Mouse | *Cd19* | Former | GGAGGCAATGTTGTGCTGC |
|  |  | Reverse | ACAATCACTAGCAAGATGCCC |
| Mouse | *Cd45* | Former | ATGGTCCTCTGAATAAAGCCCA |
|  |  | Reverse | TCAGCACTATTGGTAGGCTCC |
| Mouse | *Cd8* | Former | CCGTTGACCCGCTTTCTGT |
|  |  | Reverse | CGGCGTCCATTTTCTTTGGAA |
| Mouse | *Gapdh* | Former | AGGTCGGTGTGAACGGATTTG |
|  |  | Reverse | TGTAGACCATGTAGTTGAGGTCA |
| Mouse | *Actb* | Former | GTGACGTTGACATCCGTAAAGA |
|  |  | Reverse | GCCGGACTCATCGTACTCC |
| Human | *ACTB* | Former | CCATCGTCCACCGCAAAT |
|  |  | Reverse | GCTGTCACCTTCACCGTTCC |
| Human | *CD132* | Former | GTGCAGCCACTATCTATTCTCTG |
|  |  | Reverse | GTGAAGTGTTAGGTTCTCTGGAG |
| Human | *CD3* | Former | CCTCTTATCAGTTGGCGTTTGG |
|  |  | Reverse | TTCAGTGACAGGTGATCCTCA |
| Human | *CD4* | Former | TGCCTCAGTATGCTGGCTCT |
|  |  | Reverse | GAGACCTTTGCCTCCTTGTTC |
| Human | *CD8* | Former | ATGGCCTTACCAGTGACCG |
|  |  | Reverse | AGGTTCCAGGTCCGATCCAG |
| Human | *CD19* | Former | GGCCCGAGGAACCTCTAGT |
|  |  | Reverse | TAAGAAGGGTTTAAGCGGGGA |
